# Gold nanoparticle-decorated reduced graphene oxide as a highly effective catalyst for the selective α,β-dehydrogenation of *N*-alkyl-4-piperidones

**DOI:** 10.3762/bjnano.17.15

**Published:** 2026-01-30

**Authors:** Brenda Flore Kenyim, Mihir Tzalis, Marilyn Kaul, Robert Oestreich, Aysenur Limon, Chancellin Pecheu Nkepdep, Christoph Janiak

**Affiliations:** 1 Institut für Anorganische Chemie und Strukturchemie, Heinrich-Heine-Universität, 40204 Düsseldorf, Germany

**Keywords:** oxidative dehydrogenation, reduced graphene oxide, supported gold nanoparticles, β-N-substituted ketones

## Abstract

Gold nanoparticles (AuNPs) supported on reduced graphene oxide (AuNPs/rGO) were demonstrated to be a highly reactive catalyst for the selective α,β-oxidative dehydrogenation (ODH) of *N*-alkyl-4-piperidones, using *N*-methyl-, *N*-ethyl- and *N*-benzyl-4-piperidone. The substrate *N*-methyl-4-piperidone represents a pharmaceutically relevant system as its reaction product *N*-methyl-2,3-dihydropyridin-4(1*H*)-one is highly valuable (>1000 €·g^−1^) in contrast to the inexpensive starting material (0.15 €·g^−1^). Various synthesis methods were employed to prepare AuNPs supported on different carbon materials, including reduced graphene oxide (rGO), activated carbon (AC), and carbon black (CB), to investigate the influence of the carbon support on the catalyst performance. As stabilizing agents for the AuNPs, citrate (Cit) and the polyoxometallate [SiW_9_O_34_]^10−^ (SiW_9_) were used. Among the tested catalysts, the rGO-supported ones, Au-Cit/rGO, Au-SiW_9_/rGO, and Au@SiW_9_/rGO exhibited the highest catalytic activity for the selective oxidation reaction despite containing the lowest gold loading. These findings highlight the exceptional performance of rGO as a support for AuNP catalysts and provide valuable insights for designing efficient Au-based systems for the dehydrogenation of β-N-substituted saturated ketones and other fine chemical applications.

## Introduction

The properties related to the high surface area of matter at the nanometric scale have led to many catalytic studies at the industrial and laboratory scale [1]. Nanocatalysis is no longer just an academic field, but a rapidly evolving field for industries wishing to develop green and sustainable processes with very high turnover numbers, turnover rates, and stabilities [2].

The development of highly efficient catalysts is of constant interest for advancements in organic chemistry, particularly in oxidation, hydrogenation, and coupling reactions. Gold nanoparticles (AuNPs) have emerged as exceptionally effective catalysts for facilitating these types of reactions [3–4]. The unique catalytic properties of AuNPs stem from their nanoscale size, which increases the surface-to-volume ratio, exposes a higher density of active sites, and induces quantum size effects that modulate the electronic structure [5]. These characteristics collectively enhance their reactivity, selectivity, and tunability, making AuNPs highly versatile in catalytic applications [5–6]. Traditional ligands such as thiols and citrates are commonly used in the synthesis of AuNPs due to their ability to control particle size, prevent aggregation, and enhance stability in solution [7]. Also, polyoxometalates (POMs) have emerged as stabilizing ligands for nanoparticles offering distinct structural and electronic advantages. They are widely utilized in various catalytic processes, including oxidation, acid–base, and photocatalysis [8–9] POMs have been extensively employed for stabilizing and decorating small metal nanoparticles, including gold nanoparticles [10–11]. POMs can act as both reducing and capping agents [12]. In 2022, Xia et al. demonstrated that among the fully occupied ([SiW_12_O_40_]^4−^, SiW_12_), monovacant ([SiW_11_O_39_]^8−^, SiW_11_), divacant ([SiW_10_O_36_]^8−^, SiW_10_), and trivacant ([SiW_9_O_34_]^10−^, SiW_9_) silicotungstate POMs, the latter trivacant species exhibits the highest Au-POM reactivity in the oxidative dehydrogenation of piperidone derivatives to the corresponding enaminones [13]. Through the oxygen atoms at its three vacant sites, SiW_9_ coordinates to gold metal atoms, forming strong interactions. Despite being coated with strong ligands that provide initial stability over time, ligand desorption, ligand exchange, or environmental factors such as pH and ionic strength can weaken the protective layer, leading to nanoparticle aggregation or structural degradation [14–15].

An important aspect of expensive noble-metal nanoparticles in heterogeneous catalysis is their separation from the reaction mixture for re-use or recycling, and also to avoid contamination of the products. The difficulty in separating small nanoparticles demands for the efficient and easy formation of a composite that can be separated by filtration and where the NPs are further stabilized against coalescence, aggregation, sintering, or Ostwald ripening under the catalytic reaction conditions. By anchoring AuNPs onto a solid support, such as metal oxides, polymers, or carbon-based materials, their stability is significantly enhanced, preventing unwanted coalescence and preserving their functional properties over extended periods [16].

Carbon-based materials such as activated carbon (AC) [17], reduced graphene oxide (rGO), and carbon black (CB) have gained considerable attention as supports due to their resistance to acid and basic environments, their tunable surface area and surface chemistry, and electrical conductivity [18–19]. To deposit metal NPs onto a carbon support, procedures such as adsorption or reduction–deposition (RD), co-precipitation**,** impregnation, and deposition precipitation (DP) are commonly employed [6,19].

Carbon-supported gold nanoparticles (Au/C) have been extensively studied for their remarkable selective catalytic performance in low-temperature oxidation reactions, including CO oxidation, alcohol oxidation, hydrocarbon oxidation, amino and thiol oxidation, and glucose oxidation [20]. However, despite their widespread use in direct oxidation processes, Au/C have been less explored in selective α,β-oxidative dehydrogenation reactions, particularly for β-N-substituted saturated ketones [19,21].

**Figure 1:**
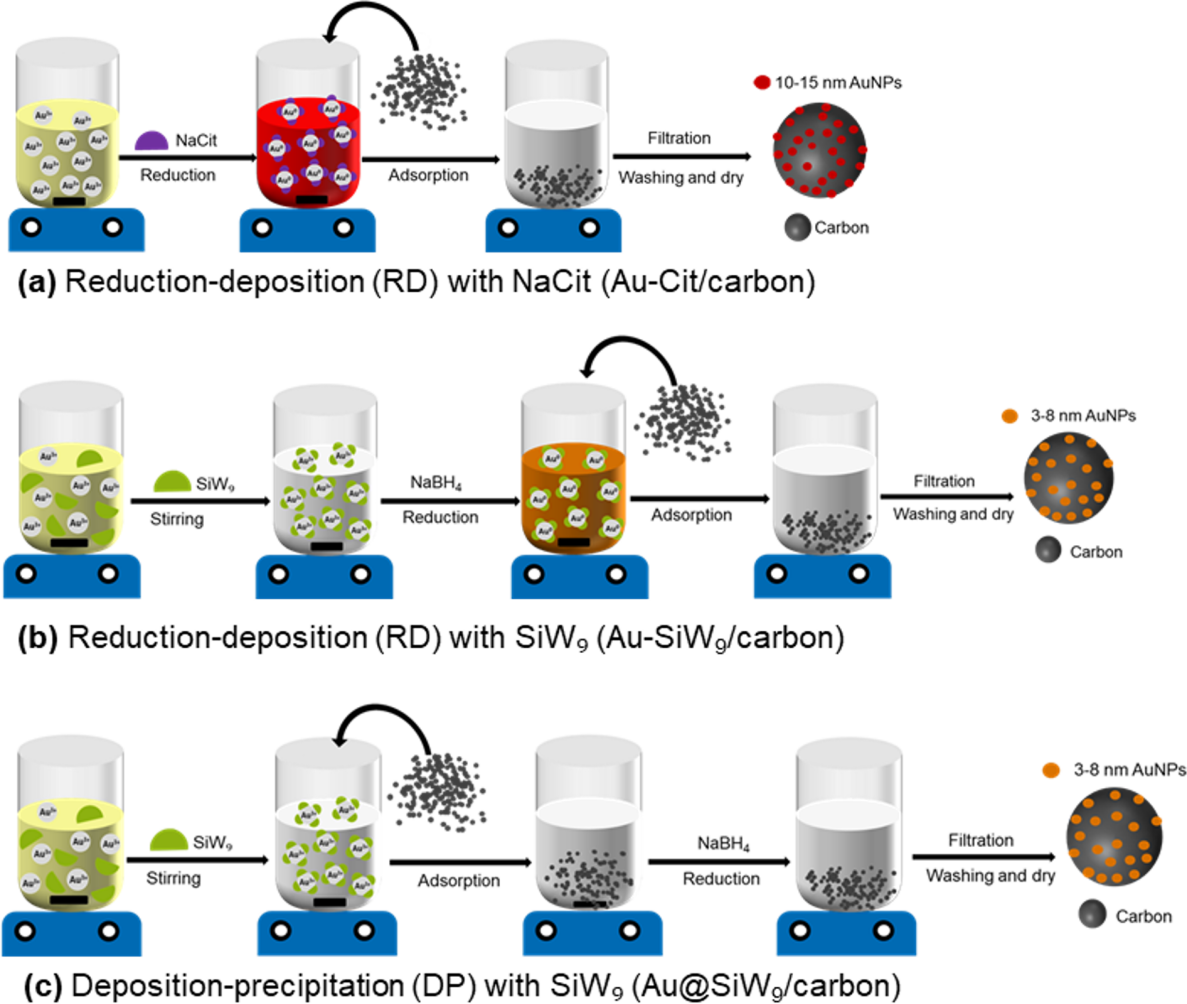
Schematic representation of the carbon-supported gold catalyst preparation through the reduction-deposition method with (a) sodium citrate, giving Au-Cit/carbon, (b) with SiW_9_, yielding Au-SiW_9_/carbon and (c) the deposition-precipitation method with SiW_9_, leading to Au@SiW_9_/carbon. In each method, the three different carbon materials activated carbon (AC), reduced graphene oxide (rGO), and carbon black (CB) were used. “Au^3+^” refers to the tetrachloridoaurate(III) anion [AuCl_4_]^–^ in the KAuCl_4_ starting material.

In this study, gold nanoparticles with an average size of 10–15 nm, stabilized by sodium citrate (NaCit), and of 3–8 nm size, stabilized by SiW_9_, and supported on the three-carbon materials AC, rGO, and CB by the RD and DP methods were synthesized as depicted in Figure 1. To assess the influence of the preparation methods and the type of the carbon supports, the catalytic activity was evaluated using the model reaction of the oxidative dehydrogenation of *N*-alkyl-4-piperidones to *N*-alkyl-2,3-dihydropyridin-4(1*H*)-ones. The relevance of this reaction lies in the fact that the product *N*-methyl-2,3-dihydropyridin-4(1*H*)-one is a valuable intermediate in medicinal chemistry. Its derivatives have demonstrated significant biological activities, including anti-cancer and anti-bacterial effects [22]. The pharmaceutical relevance of this compound is further underscored by its high market value (>1000 €·g^−1^), in stark contrast to the low cost of the starting material.

The novelty of this work lies primarily in the use of reduced graphene oxide (rGO) as an advanced carbon-based support, which provides unique electronic interactions with gold nanoparticles, and its comparison to AC and BC. The rGO support was not tested in the work by Xia et al. [13] where only activated carbon was used. Strong Au–rGO interfacial coupling should facilitate charge transfer between the metal and the support, enhancing the activation of molecular oxygen and promoting the oxidative dehydrogenation (ODH) pathway. This synergistic interaction should not only improve the intrinsic catalytic activity but also allow the catalyst to achieve high efficiency and selectivity with an exceptionally low gold loading.

## Results and Discussion

### Description and characterization of the carbon supports

The morphological and textural properties of the carbon supports are critical for their performance as catalysts in AuNP-supporting systems. To assess these properties, scanning electron microscopy (SEM) and nitrogen gas sorption surface area analysis using the Brunauer–Emmett–Teller (BET) theory were employed to evaluate the surface structure, porosity, and overall texture of the materials.

**Figure 2:**
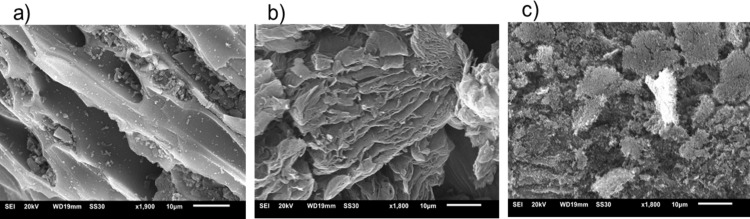
SEM images (10 µm scale) of (a) AC, (b) rGO, and (c) CB.

The SEM image in Figure 2a reveals the typical structure of AC, characterized by a heterogeneous, rough surface with lamellar layers and pronounced macroporosity. The rGO carbon (Figure 2b) exhibits a structure composed of thin, wrinkled, and sheet-like layers, characteristic of exfoliated graphene-based materials. The morphology of CB (Figure 2c) is formed of highly aggregated small particles.

**Figure 3:**
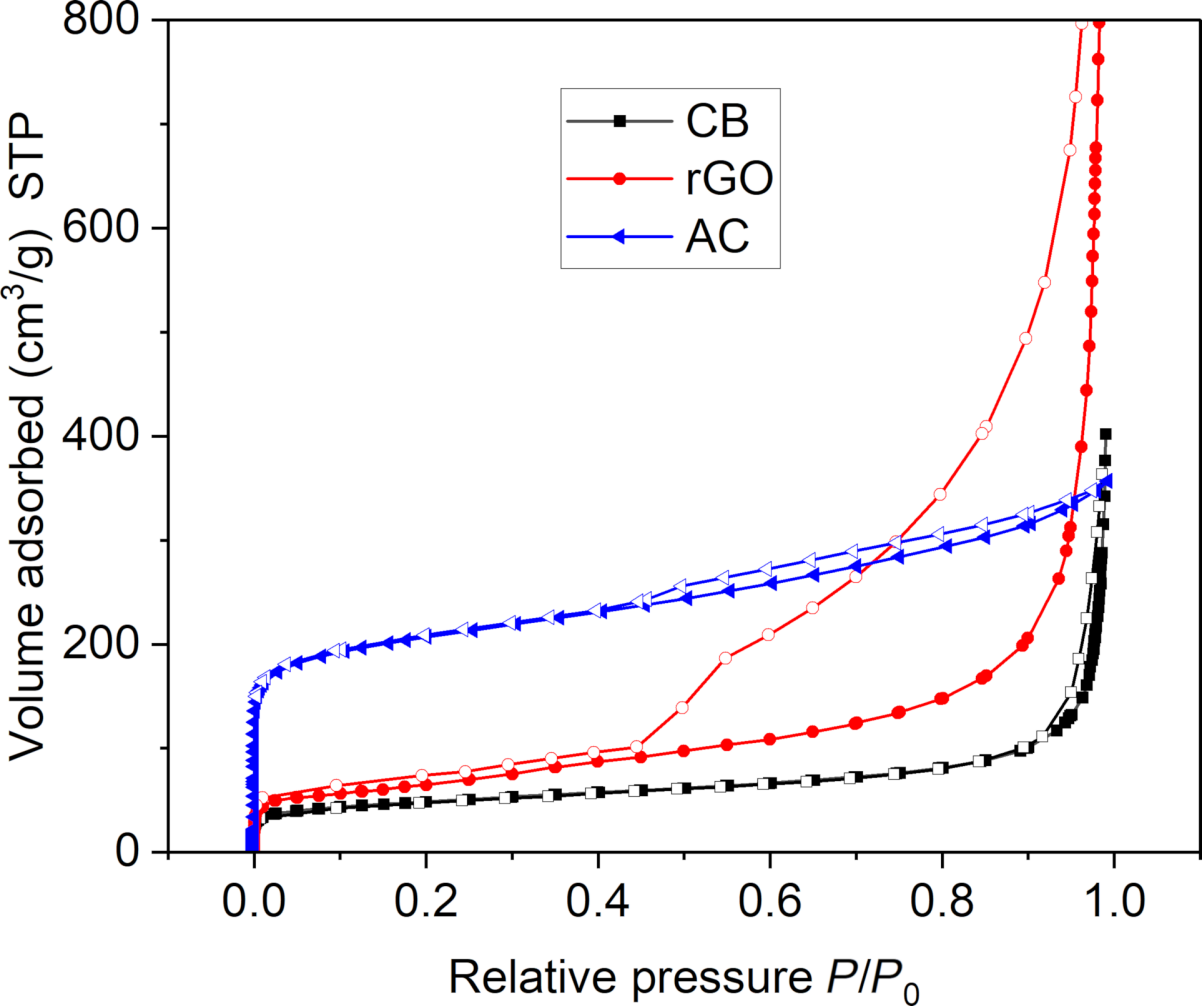
Nitrogen sorption isotherms of the three carbon materials at 77 K (filled symbols: adsorption, empty symbols: desorption).

The nitrogen sorption isotherms and their hysteresis loops in Figure 3 supplement the SEM analysis of the three carbon materials in terms of specific BET surface area, and pore volume and size. AC features a reversible almost type-I isotherm, given by microporous materials with narrow micropores around 1 nm, with a small type-II contribution at larger *P*/*P*_0_ and a small H4 hysteresis loop, which is given by the mesoporous part of AC. rGO has a type-II isotherm due to unrestricted monolayer–multilayer adsorption up to high *P*/*P*_0_; the multilayer absorption appears to increase without limit when *P*/*P*_0_ reaches 1. The wide H3 hysteresis loop is typically given by non-rigid aggregates of platelets as seen in rGO (cf. Figure 2b). CB also gives a combination of a type-I and a type-II isotherm, albeit with an overall small uptake and a very narrow H3 hysteresis loop. The lower limit of this H3 loop is located at the cavitation-induced *P*/*P*_0_, which is at a high value of 0.9 in CB and at 0.45 in rGO [23].

Among the three carbon materials, activated carbon exhibited the highest BET surface area (751 m^2^·g^−1^, Table S1) and an intermediate total pore volume (0.81 cm^3^·g^−1^). Its pore diameters are largely below 2 nm and indicate a predominantly microporous structure (Figure S1a, Supporting Information File 1), which may limit mass transport and hinder catalytic performance in reactions requiring easy access to active sites. Reduced graphene oxide has a moderate BET surface area (231 m^2^·g^−1^), but the highest total pore volume (2.03 cm^3^·g^−1^). The pore diameters extend into the mesoporous region (2–50 nm) for a large fraction of the pores (Figure S1b, Supporting Information File 1). This combination suggests a more open and accessible porous network, facilitating better mass transport and diffusion of reactants and products to and from the active sites. Carbon black shows the lowest BET surface area (167 m^2^·g^−1^) and total pore volume (0.57 cm^3^·g^−1^). Most of the pores are again in the microporous region (<2 nm) (Figure S1c, Supporting Information File 1), concomitant with a less accessible structure, which could limit catalytic performance.

Although activated carbon has the highest surface area, its low microporous nature may limit accessibility to active sites. In contrast, rGO presents a balanced combination of a reasonably high surface area, a larger pore diameter, and the highest pore volume. These features provide a more favorable environment for mass transport, reactant diffusion, and nanoparticle dispersion, making rGO a more effective catalyst support, so we expect a better catalytic activity with rGO.

In addition to the morphological and textural properties of carbon supports, which have an impact on the dispersion of metal nanoparticles and the overall efficiency of the catalytic process, the presence and distribution of functional groups is also a crucial factor in the choice of the carbon support. Several studies have demonstrated that the amount of surface oxygen-containing functional groups strongly influences catalytic activity [24–25]. AC contains a significant number of functional groups, such as carboxyl, hydroxy, and phenol groups, which enhance its interaction with metal nanoparticles and improve catalytic performance [26–27], rGO possesses a higher amount of oxygen functional groups than AC [27]. In contrast, CB has fewer oxygen-containing functional groups, making it more chemically inert with weaker metal–support interactions [28]. Based on these findings [27–28], the abundance of oxygen-containing functional groups among the studied carbon supports follows the order rGO > AC > CB, making rGO probably the most active and efficient support for NPs.

### Synthesis and characterization of carbon-supported gold nanoparticles

The synthesis of the Au-Cit/carbon and of the Au-SiW_9_/carbon composites was carried out by the RD method as schematically depicted in Figure 1a,b. This method involved the synthesis of AuNPs in a colloidal solution, using sodium citrate both as a reducing and stabilizing agent (Figure 1a) or the silicotungstate polyoxometallate SiW_9_ as a stabilizer with NaBH_4_ as the reducing agent (Figure 1b). The preformed nanoparticles were then deposited onto the carbon material. The synthesis of the Au@SiW_9_/carbon composites was done by the DP method as shown in Figure 1c. The POM salt (SiW_9_), the gold precursor (KAuCl_4_), and the carbon material were combined in a solution–dispersion, and the reduction was induced with NaBH_4_.

#### Synthesis of citrate-coated AuNPs on carbon

The citrate-coated AuNPs (Au-Cit) were synthesized by the Turkevich method, one of the most widely used bottom-up techniques. In this process, the gold salt KAuCl_4_ is reduced by sodium citrate (NaCit) when the aqueous solution is heated to boiling. Upon adding sodium citrate to the tetrachloridoaurate(III) solution, the solution initially becomes decolorized as gold(III) is reduced to gold(I). After a few minutes, the solution turns violet, signaling the formation of AuNPs, which then transition to a ruby red color as the AuNPs disperse. This result is a colloidal gold nanoparticle suspension.

**Figure 4:**
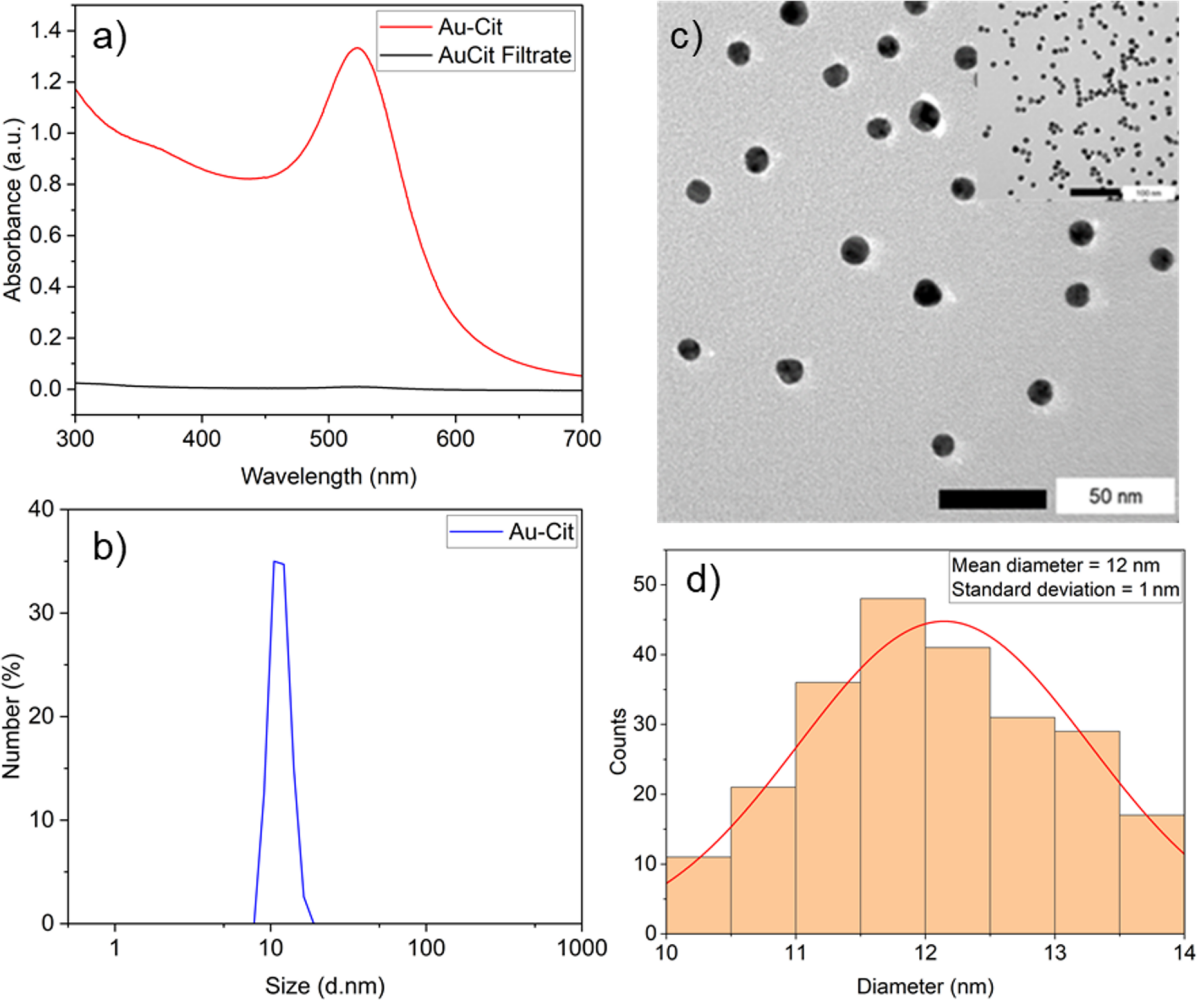
(a) Localized surface plasmon resonance absorption band of AuNPs present in the dispersion (Au-Cit) and the spectrum of the filtrate after AuNP deposition on carbon; (b) DLS of Au-Cit. (c, d) TEM images of Au-Cit and the corresponding particle size distribution histogram. Conditions: *n*_KAuCl4_ = 53 µmol, *n*_NaCit_ = 319 µmol, and *T* = 100 °C.

The presence of AuNPs was confirmed by the appearance of a localized surface plasmon resonance (LSPR) band in the visible wavelength range, with a maximum absorbance at λ_max_ = 519 nm (Figure 4a). The hydrodynamic radius (HD) by number-weighted distribution of dynamic light scattering (DLS) measurements was 12 nm (Figure 4b). Transmission electron microscopy (TEM) analysis showed spherical and well-dispersed nanoparticles and their particle size distribution (Figure 4c), based on the measurements of approximately 200 nanoparticles, yielded an average diameter of 12 nm with a standard deviation of ±1 nm (Figure 4d), which agrees with the size obtained by DLS.

**Figure 5:**
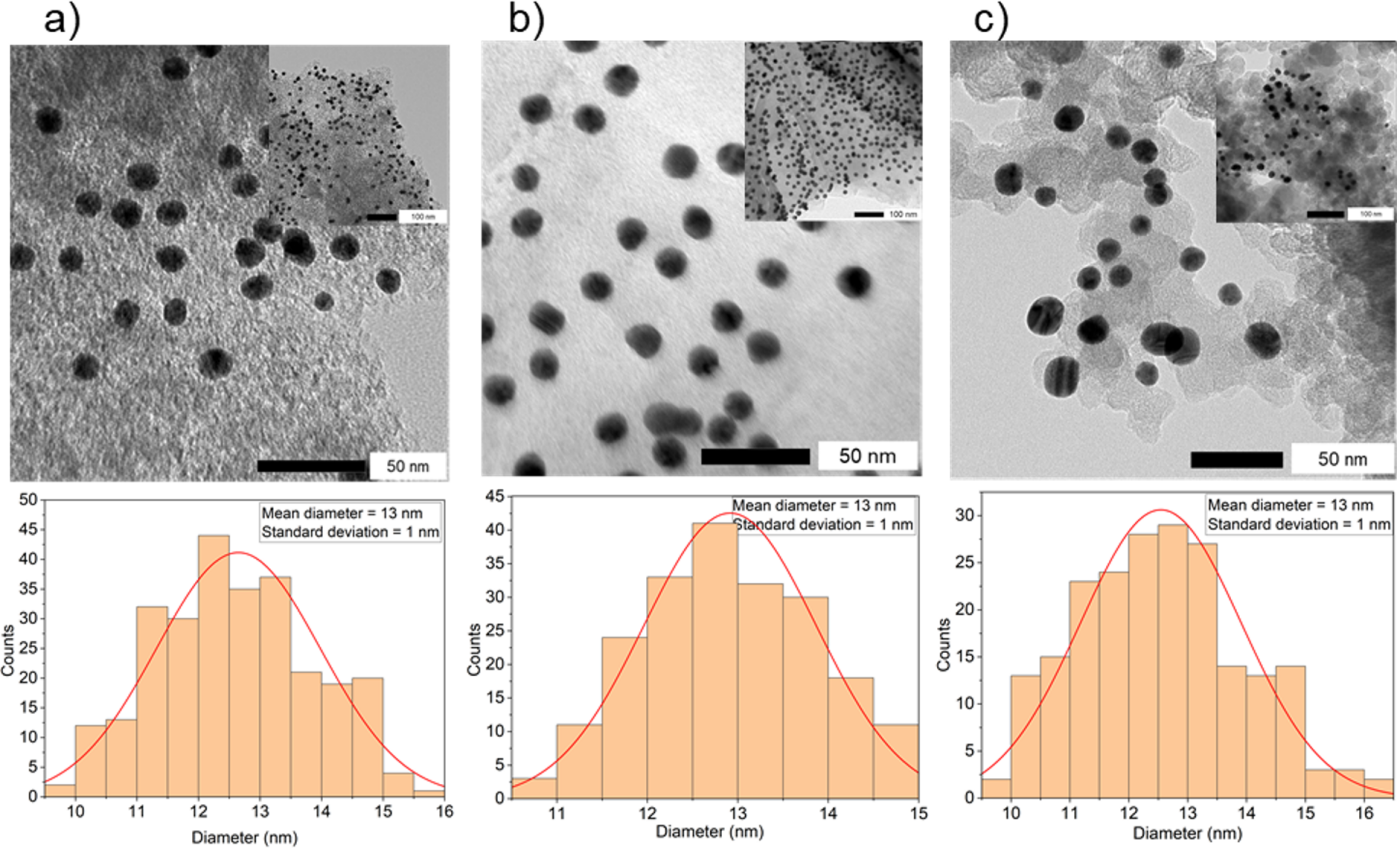
TEM images of (a) Au-Cit/AC, (b) Au-Cit/rGO, and (c) Au-Cit/CB, with the inserts showing a wider area and their corresponding particle size distribution histograms below each of them.

Immediately after synthesizing three batches of the citrate-coated AuNPs, the three different carbon materials AC, rGO, and CB were individually introduced into separate batches (Figure 1a). In all cases, this resulted in complete discoloration of the dispersion to a clear solution with a black precipitate, indicating the successful deposition of AuNPs onto the carbon supports. This was further confirmed by spectrophotometric analysis of the filtrate, which showed the disappearance of the LSPR band (Figure 4a). Subsequently, the successful AuNP deposition on the carbon materials was validated through TEM imaging of Au-Cit/AC (Figure 5a), Au-Cit/rGO (Figure 5b), and Au-Cit/CB (Figure 5c). Upon deposition, the average AuNP particle sizes slightly grew to 13 nm within a range of 10–16 nm determined by TEM images and the corresponding distribution histograms. This growth was probably due to continuous agitation during colloid immobilization.

Noteworthy, the AuNPs are evenly distributed on rGO but occupy only certain areas on AC and especially on CB, thereby leaving large sections of the CB surface without AuNPs. This is in agreement with the fewer oxygen-containing functional groups leading to less metal–support interactions on CB [15].

Several factors facilitate effective AuNP anchoring and homogeneous dispersion on rGO. First, rGO is only partially reduced, as confirmed by XPS, retaining oxygen-containing functional groups (C–O, C=O, –OH) that can form hydrogen bonds or coordination interactions with citrate ligands and Au species [29–30]. These localized polar sites promote adsorption. Second, charge inhomogeneities on rGO arising from structural defects, residual oxygenated patches, and edge terminations create domains that enhance electrostatic attraction of AuNPs [31]. These combined effects explain the more homogeneous AuNP dispersion on rGO compared to AC or CB, which contain fewer functional groups and more inert graphitic surfaces [32]. AC often contains micropores that can trap some nanoparticles, resulting in non-uniform distribution or partial aggregation [33]. CB, being highly graphitized and relatively inert, lacks sufficient anchoring groups; thus, AuNPs are mostly adsorbed through weak van der Waals forces, leading to poorer dispersion [33].

#### Synthesis of SiW_9_-coated AuNPs on carbon

**Figure 6:**
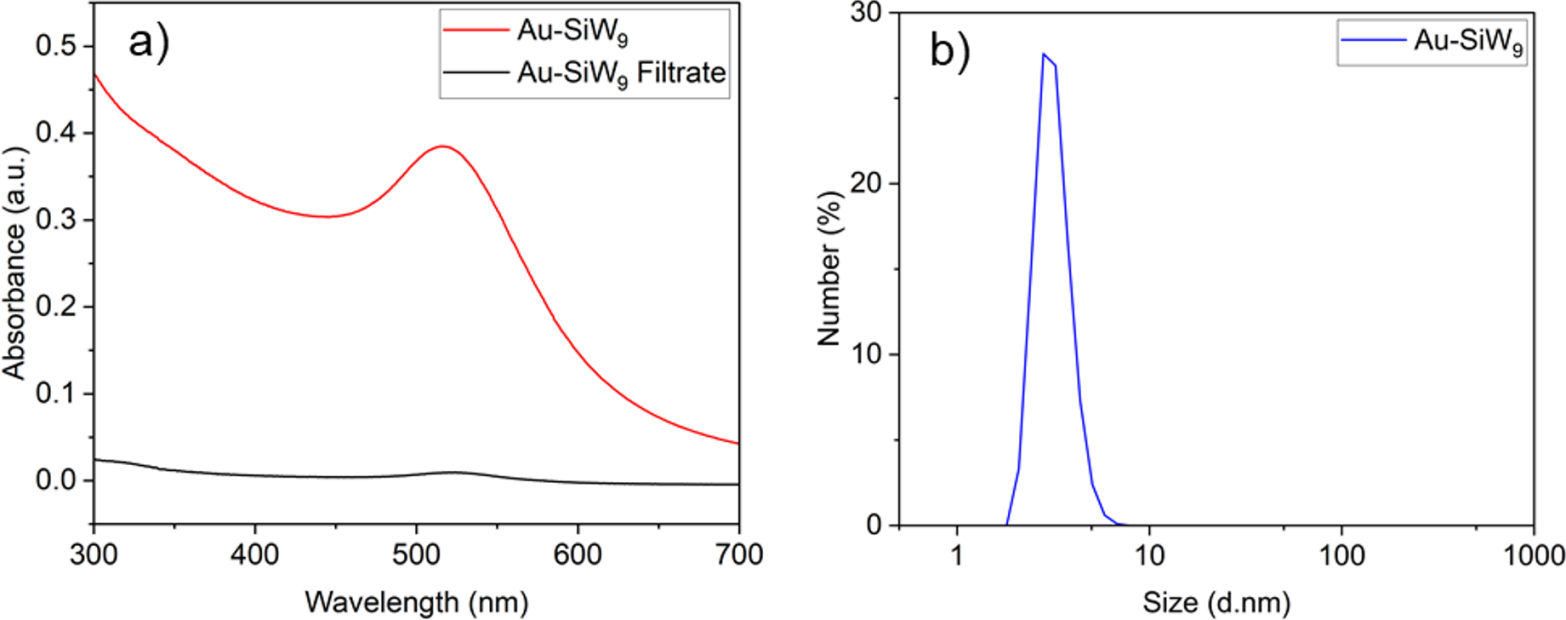
(a) Plasmon resonance absorption band of AuNPs present in the dispersion (Au-SiW_9_) and the spectra of the filtrate after AuNPs deposition on AC, rGO, and CB; (b) DLS of Au-SiW_9_ dispersion. Conditions: *n*_KAuCl4_ = 15 µmol, *n*_SiW9_ = 15 µmol, *n*_NaBH4_ = 150 µmol, and *T* = 2 °C.

Unlike the synthesis of Au-Cit, which takes place under hot conditions, the synthesis of Au-SiW_9_ is performed in the cold at 2 °C to prevent the isomerization of the POM. First, the sodium salt of SiW_9_ (Na_10_SiW_9_O_34_) was synthesized following a well-established protocol [34] and characterized by powder X-ray diffraction (PXRD) (see Supporting Information File 1). An ice-cold solution of sodium borohydride (NaBH_4_) was added dropwise to an aqueous solution containing KAuCl_4_ and the sodium salt of SiW_9_, resulting in the formation of an orange-brown dispersion (Figure 1b). UV–vis spectroscopy revealed the characteristic LSPR band at 508 nm, confirming the presence of gold nanoparticles (Figure 6a). Compared to Au-Cit with λ_max_ = 519 nm, the blueshift observed for Au-SiW_9_ indicated the formation of smaller nanoparticles, which was further confirmed by DLS analysis, showing an average particle diameter of approximately 4 nm (Figure 6b).

**Figure 7:**
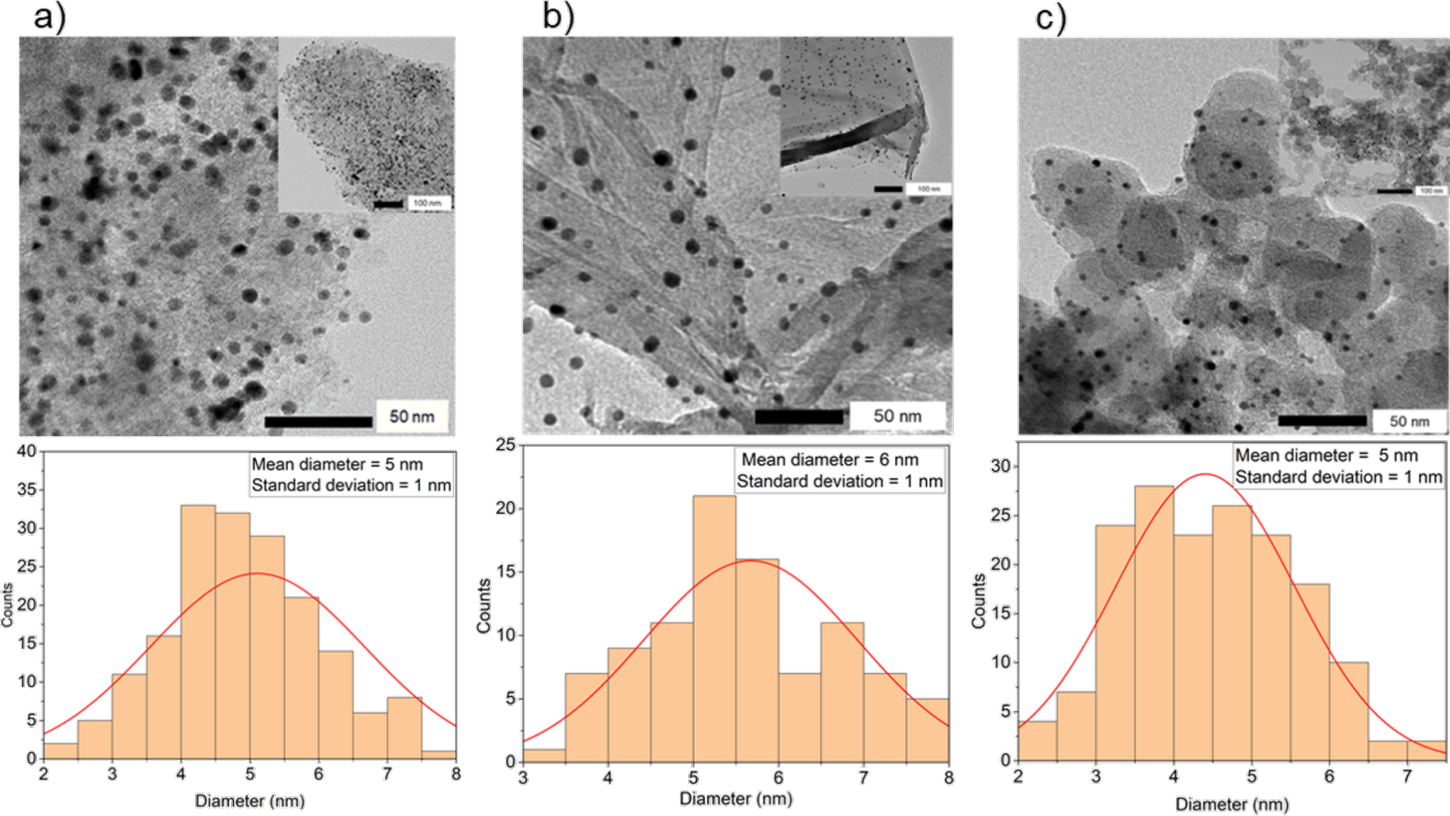
TEM images of (a) Au-SiW_9_/AC, (b) Au-SiW_9_/rGO, and (c) Au-SiW_9_/CB with the inserts showing a wider area and their corresponding particle size distribution histograms. For higher resolution images see Supporting Information File 1, Figure S2.

Following the formation of the colloidal suspension of Au-SiW_9_, the carbon supports (AC, rGO, and CB) were added (Figure 1b). This addition resulted in a slight discoloration of the dispersion, transitioning from brown-orange to colorless within a few minutes. This change indicates the successful deposition of Au-SiW_9_ onto the carbon supports. The successful deposition was further confirmed through UV–vis analysis of the filtrate obtained after the filtration step, which revealed the disappearance of the characteristic LSPR band at 508 nm (Figure 6a). The TEM images of the synthesized composites, Au-SiW_9_/AC (Figure 7a), Au-SiW_9_/rGO (Figure 7b), and Au-SiW_9_/CB (Figure 7c), revealed small, spherical, and well-dispersed particles. The particle size was between 2 and 8 nm with an average of 5–6 nm and a standard deviation of ±1 nm, as determined from their corresponding distribution histograms (Figure 7). The Au-SiW_9_/AC composite had already been synthesized by Xia et al. [13] and our results of particle size and distribution closely match their outcomes, confirming the successful deposition and comparable dispersion of gold nanoparticles on the activated carbon support.

#### Synthesis of Au@SiW_9_/AC, Au@SiW_9_/rGO, and Au@SiW_9_/CB

The DP method is the most widely used procedure to support gold nanoparticles on metal oxides [6]. The synthesis involves dissolving the POM salt (SiW_9_) and the gold precursor (KAuCl_4_) in water in an ice bath, dispersing the carbon material (AC, rGO, CB) in the solution, and allowing POM salt and gold precursor to adsorb onto the carbon surface or in the pores; then, the reduction with NaBH_4_ is induced to form Au@SiW_9_/AC, Au@SiW_9_/rGO, Au@SiW_9_/CB. Unlike the RD method, where the reduction of the tetrachloridoaurate(III) ions to metallic gold occurs in solution and is observable through a color change before the addition of the carbon material, the DP synthesis method induces the reduction within the pores or on the surface of the carbon supports (Figure 1c). As a result, no color change is perceptible when the amount of carbon was sufficient to capture the gold precursor from solution. Hence, DLS analysis of the AuNPs will neither be possible. Only TEM analysis can be used to characterize the gold nanoparticles within the carbon matrix.

**Figure 8:**
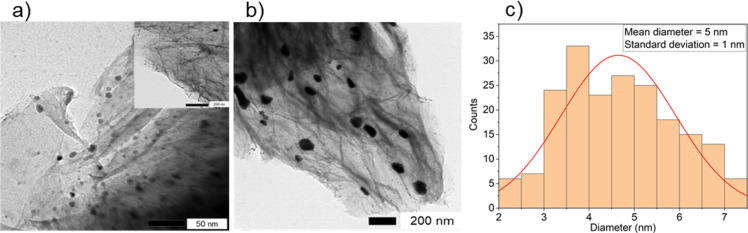
(a, b) TEM images of Au@SiW_9_/rGO at two different magnifications and (c) the corresponding particle size distribution histogram.

Upon examining the TEM images at 50 nm magnification of the Au@SiW_9_/rGO system synthesized via DP (Figure 8a), it is evident that the AuNPs are less dense and less discernible than those obtained with the RD method (cf. Figure 7a). The average particle size is 5 nm in a range between 2 and 7 nm, as determined from their corresponding distribution histograms (Figure 8c). There are also strongly aggregated AuNPs seen in many sections of the Au@SiW_9_/rGO composite (Figure 8b). For Au@SiW_9_/AC and Au@SiW_9_/CB, such strong aggregation is all what is seen for the Au@SiW_9_ particles (Supporting Information File 1, Figure S2). According to the literature, these are indeed aggregates and not large individual particles [6,35]. These observations suggest that, during the adsorptive pre-deposition process, the anionic [AuCl_4_]^−^ precursor will adsorb at different regions or smaller pores than the larger SiW_9_ anions intended for stabilization. When the reduction is induced, there will not be enough POM anions in the vicinity to prevent aggregation. Uneven precursor distribution can lead to localized high concentrations of Au species, resulting in less controlled nucleation and the formation of larger clusters. Surface roughness and heterogeneity of the support influence the size and distribution, leading to increased aggregation of AuNPs [36–37]. Evidently, rGO with its higher amount of mesopores and functional oxygen groups is the only carbon material among the three that still allows for the formation of a significant fraction of non-aggregated AuNPs.

**Figure 9:**
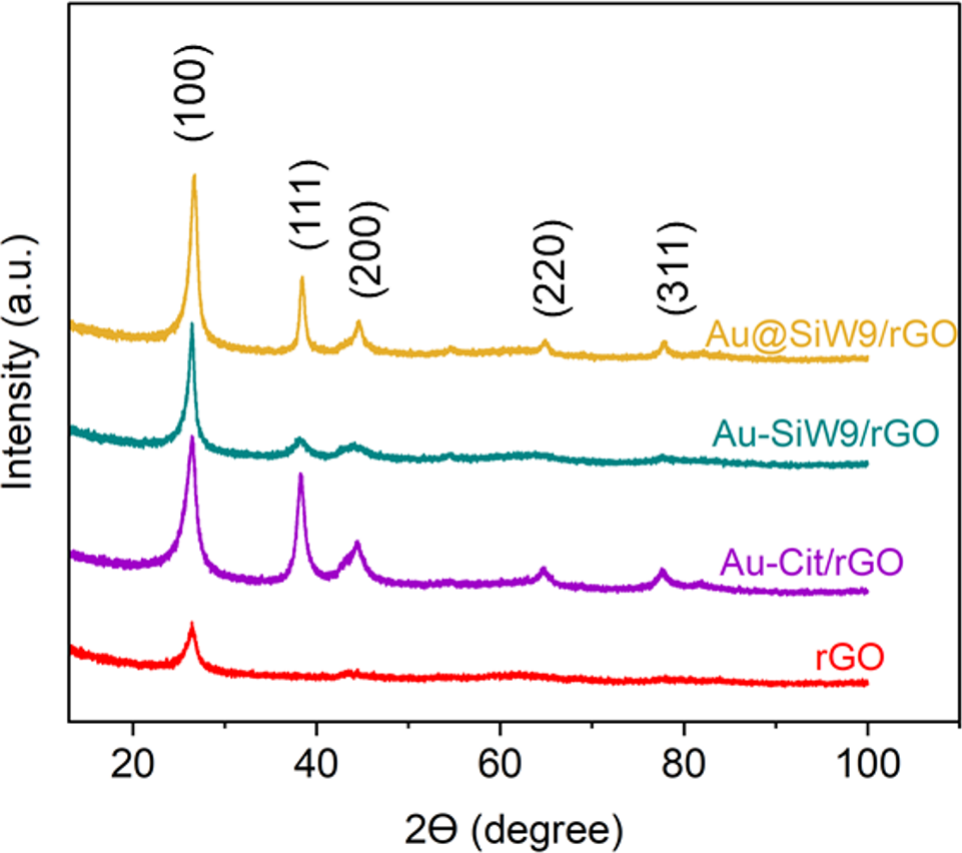
PXRD patterns of crystalline gold nanoparticles deposited on rGO.

The PXRD patterns of the AuNPs deposited on rGO carbon by the different methods show the crystallinity of the AuNPs, with four distinct diffraction peaks at 38.4° (111), 44.5° (200), 64.8° (220), and 77.8° (311) (Figure 9), which correspond to the face-centered cubic (fcc) gold lattice [38]. The first distinct diffraction peak at 2θ = 25.5° is attributed to the (100) reflection of rGO [39].

The crystallite size of the gold nanoparticles (AuNPs) is determined by X-ray diffraction (XRD) analysis using the Scherrer equation (Equation 1) below. The shape factor *k*, often referred to as the Scherrer constant, is influenced by several factors, including the crystallites’ shape, degree of size uniformity, and the nature of the diffraction peak. For nanoparticles with a spherical shape and cubic symmetry, a commonly adopted value for *K* is 0.94 [40]. *D* represents the average crystallite size, λ is the X-ray wavelength (0.1542 nm), β corresponds to the full width at half maximum (FWHM) of the diffraction peak (in radians), and θ is the Bragg angle. The average crystallite dimension was found to be approximately 14 nm for the catalyst Au-Cit/rGO and 7 nm for Au-SiW_9_/rGO and Au@SiW_9_/rGO.


[1] $$D=\frac{k\lambda }{\beta \cos\left(\theta \right)}$$

The average crystallite size was determined to be approximately 15 nm for the Au-Cit/rGO catalyst, and around 7 nm for both Au-SiW_9_/rGO and Au@SiW_9_/rGO. These values correlate with the particle size distributions observed in the TEM histogram analysis. The slight variation in measured sizes can be attributed to limitations of the instrumentation, the presence of structural defects, the complexity of signal–sample interactions, and background noise [41]. As a result, it can be challenging to distinguish peak broadening caused specifically by crystallite size from that induced by other contributing factors [42–43]. Nevertheless, the Scherrer equation remains a valuable tool for estimating the average size of nanoparticles based on X-ray diffraction data.

To verify the gold loading in the Au-SiW_9_/rGO composite, a carefully weighed aliquot was dissolved in a microwave digestion system using conc. HNO_3_ (70%) and diluted HCl (5%) for atomic absorption spectroscopy (AAS). The measured gold concentration obtained by AAS (4.1 mg·L^−1^) is close to the theoretical value (4.22 mg·L^−1^) based on the assumption of quantitative gold deposition from the gold precursor on the rGO together with SiW_9_. Thus, gold incorporation into the Au-SiW_9_/rGO composite occurred as expected, and no significant gold metal loss took place during preparation or handling. AAS thus gave a gold loading of 4.1 wt % of Au on SiW_9_/rGO.

**Figure 10:**
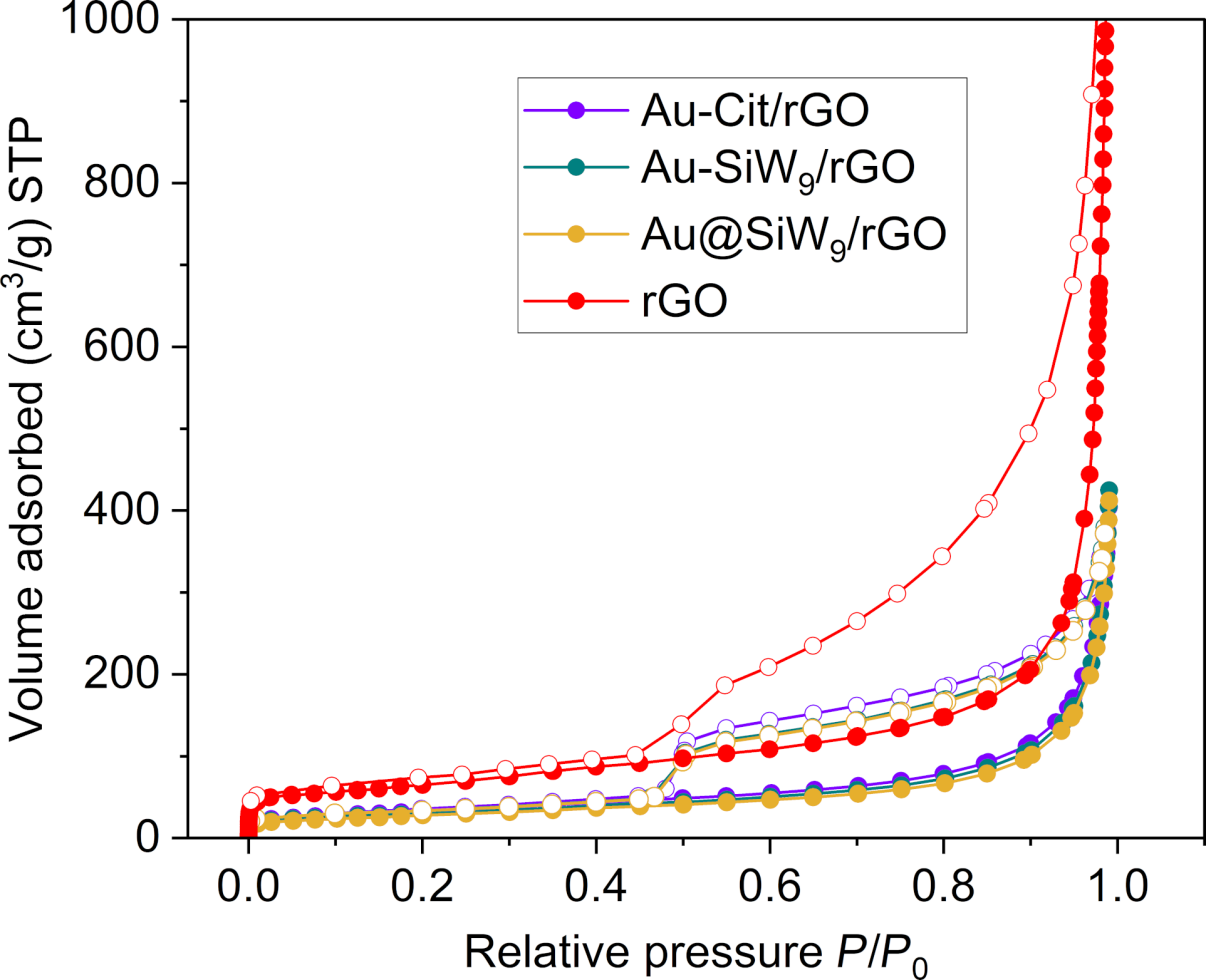
Nitrogen sorption isotherms of the AuNP/rGO composites at 77 K (filled symbols: adsorption, empty symbols: desorption).

After the deposition of AuNPs, the BET surface area and total pore volume of the composites showed significantly lower values of less than 50% compared to those of neat rGO (Supporting Information File 1, Table S2, Figure 10). Considering the thin-layer structure and small pore size of rGO, the Au nanoparticles are likely too large to enter and occupy the porous network and are instead predominantly deposited on the surface. In part, the observed reduction in specific (i.e., mass-based) surface area can be attributed to the additional ≈10 wt % mass of the Au nanoparticles with the additional mass of their citrate or SiW_9_ conjugate, which are non-porous, but add to the mass of the composite and thereby lead to an inherently lower specific surface area. However, even when assuming a mass fraction of 80 wt % of carbon in the composites, the surface areas and porosities should lie at ≈80% of those of neat rGO. The even lower values of less than 50% are therefore explained by pore-blocking phenomena through the deposition of the AuNPs.

**Figure 11:**
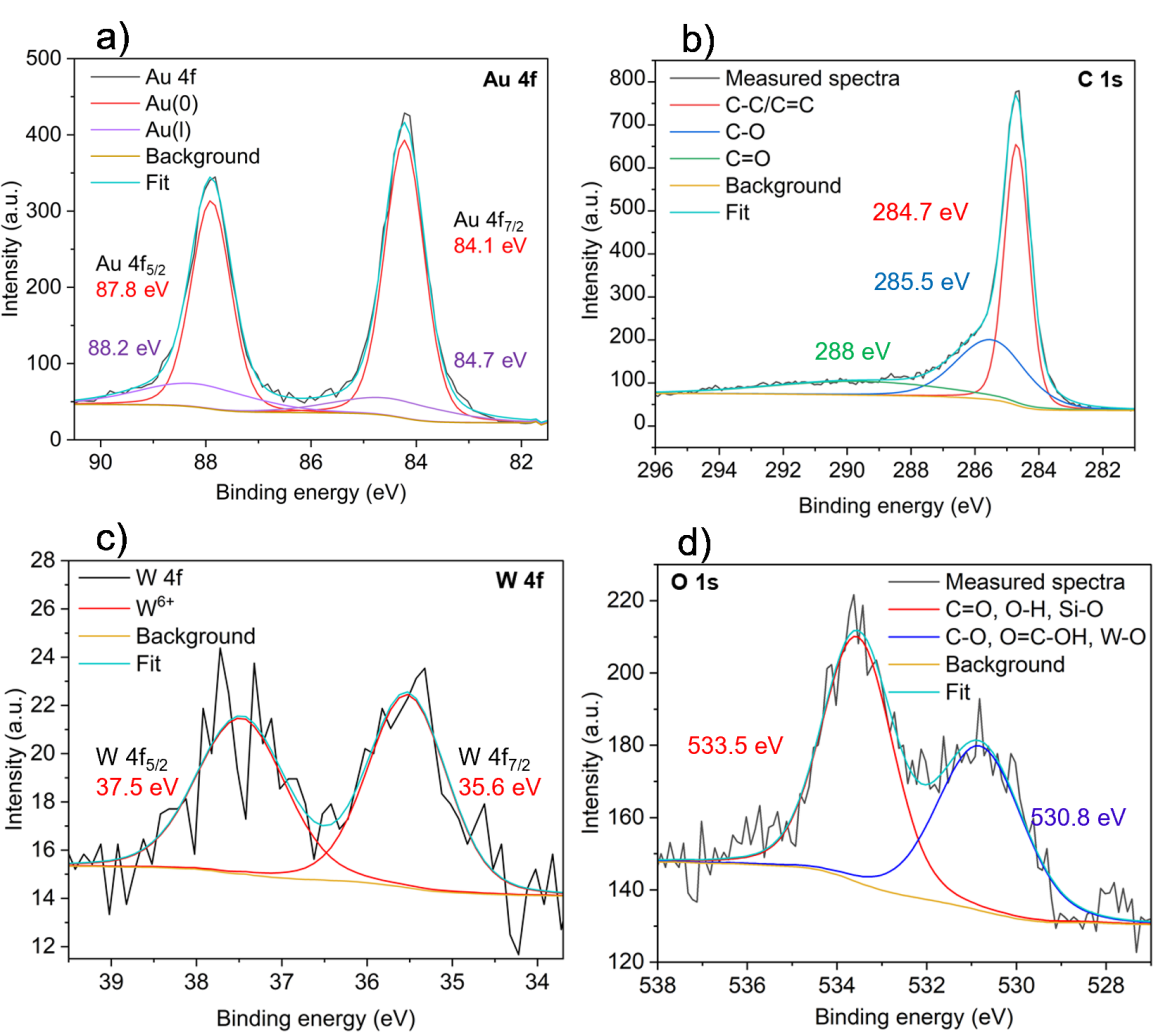
Deconvoluted XPS spectra of the (a) Au 4f, (b) C 1s, (c) W 4f, and (d) O 1s regions of the Au-SiW_9_/rGO composite.

The oxidation states of gold and the surface functionalities of rGO were investigated by X-ray photoelectron spectroscopy (XPS) in the composite Au-SiW_9_/rGO. The Au 4f region (Figure 11a) shows two dominant peaks at 84.1 eV (Au 4f_7/2_) and 87.8 eV (Au 4f_5/2_), which correspond to metallic Au^0^, which is the initial catalytically active form in oxidative dehydrogenation (ODH) reactions. A minor component at slightly higher binding energies (88.2 and 84.7 eV) is attributed to Au^+^ species resulting from surface interaction with oxygenated groups of rGO. The predominance of Au^0^ demonstrates that the active sites mainly consist of metallic gold nanoparticles.

The C 1s spectrum (Figure 11b) displays deconvoluted peaks at 284.7 eV (C–C/C=C), 285.5 eV (C–O), and 288 eV (C=O), confirming the oxygen-containing functionalities in rGO. These residual groups promote strong Au–support interactions, improving nanoparticle dispersion and stability [44]. The W 4f region (Figure 11c) exhibits two peaks at 35.6 eV (W 4f_7/2_) and 37.5 eV (W 4f_5/2_), characteristic of W^6+^ in the polyoxometalate framework [44]. This confirms that the [SiW_9_O_34_]^10−^ stabilizer remains largely intact and continues to contribute to AuNP stabilization. The O 1s spectrum (Figure 11d) reveals peaks at 530.8 and 533.5 eV, corresponding to [SiW_9_O_34_]^10−^ lattice oxygen and rGO surface oxygenated groups. The broadness of the higher-energy peak suggests contributions from C–O and O=C–OH groups at lower binding energies, C=O and C–OH groups at higher binding energies, and minor contributions from W–O and Si–O bonds of the polyoxometalate stabilizer. These oxygen functionalities provide anchoring sites for AuNPs, enhancing their dispersion and stability on the rGO surface.

No distinct Si 2p peak was observed, which can be attributed to the low surface concentration of Si and attenuation by the overlying AuNPs and carbon layers. Similar behavior has been reported in other Au–polyoxometalate systems where the W signal dominates and the Si 2p peak is below detection limits [45].

Collectively, these XPS results confirm that gold is predominantly metallic (Au^0^) and the rGO support contains functional groups that promote strong metal–support interaction, ensuring excellent nanoparticle dispersion and catalyst stability.

#### Catalytic test of the carbon-supported AuNPs

**Scheme 1:**
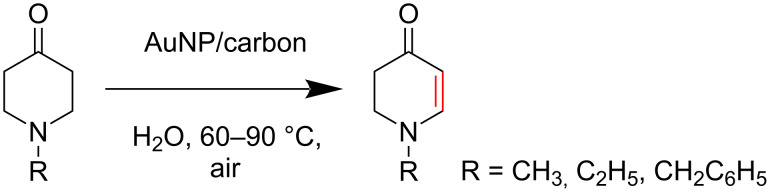
α,β-oxidative dehydrogenation of *N*-methyl-, *N*-ethyl-, and *N*-benzyl-4-piperidone to *N*-alkyl-2,3-dihydropyridin-4(1*H*)-ones with gold catalysts and aerial oxygen in aqueous dispersions.

To evaluate the efficiency of the synthesized AuNP–carbon composites, we selected the relevant oxidative dehydrogenation of *N*-methyl-, *N*-ethyl-, and *N*-benzyl-4-piperidone to the respective *N*-methyl-2,3-dihydropyridin-4(1*H*)-ones as a model reaction (Scheme 1). This transformation is an example of the synthesis of β-*N*-substituted α,β-unsaturated ketones and requires catalysts with strong oxidizing capabilities, such as gold-based catalysts.

The nine carbon-supported gold nanoparticle materials synthesized in this study were evaluated under identical conditions, at first for the *N*-methyl-4-piperidone (Table 1), focusing on conversion, selectivity, and yields. Blank tests demonstrated that the neat carbon supports (AC, rGO, CB) without gold loading exhibited no catalytic activity. Conversion rates and yields of the reaction were determined by gas chromatography (GC) using calibration curves of the starting material and the product (see Supporting Information File 1).

**Table 1:** Catalytic results in the selective ODH of *N*-methyl-4-piperidone to *N*-methyl-2,3-dihydropyridin-4(1*H*)-one.^a^

Entry	Catalyst	Au (wt %)^b^	Catalyst (mg)^c^	Au/substrate^d^	Time (h)	Conversion (%)^e^	Yield (%)^f^

1	Au-Cit/AC	1.04	100	0.021	8	100	90
2	Au-Cit/rGO	10.44	10	0.021	7	100	98
3	Au-Cit/CB	2.08	50	0.021	8	60	48

4	Au-SiW_9_/AC^g^	0.42	100	0.0085	8	100	88
5	Au-SiW_9_/rGO^g^	4.22	10	0.0085	6	100	98
6	Au-SiW_9_/CB^g^	0.84	50	0.0085	8	42	22

7	Au@SiW_9_/AC^h^	0.42	100	0.0085	8	85	62
8	Au@SiW_9_/rGO^h^	4.22	10	0.0085	7	100	98
9	Au@SiW_9_/CB^h^	0.84	50	0.0085	8	23	11

^a^Reaction conditions: 30 mg, 0.25 mmol of *N*-methyl-4-piperidone, 2 mL of water, open air 1.013 bar, temperature 60 °C. ^b^Weight fraction of Au in the composite, calculated by assuming quantitative uptake of the AuNPs or the gold precursor onto the carbon material. ^c^Amount of applied composite in the catalysis. The amount was chosen so as to achieve the same molar fraction of Au in the reaction mixture of the three Au-X/carbon catalysts. ^d^Molar ratio of gold to starting material in the reaction mixture = mol Au/mol piperidone. ^e^Conversion was determined from the molar concentration of the starting material before the reaction minus the starting material after the reaction divided by the concentration of the starting material before (×100%). The concentration was derived from the GC signal area (Figure S8, Supporting Information File 1) with the calibration curves (Figure S7a). ^f^Yield was determined from the molar amount of the product divided by the used molar amount of the starting material (×100%). The molar amount of the product was determined from the GC signal area (Figure S8) with the calibration curve (Figure S7b, Supporting Information File 1). ^g^Au-SiW_9_/carbon derived from dropwise addition of a NaBH_4_ solution to an aqueous solution of KAuCl_4_ and the sodium salt of SiW_9_ to which then the carbon support was added. ^h^Au@SiW_9_/carbon refers to the DP method where the POM salt and gold precursor were adsorbed onto the carbon surface or in the pores and then reduced with NaBH_4_.

**Figure 12:**
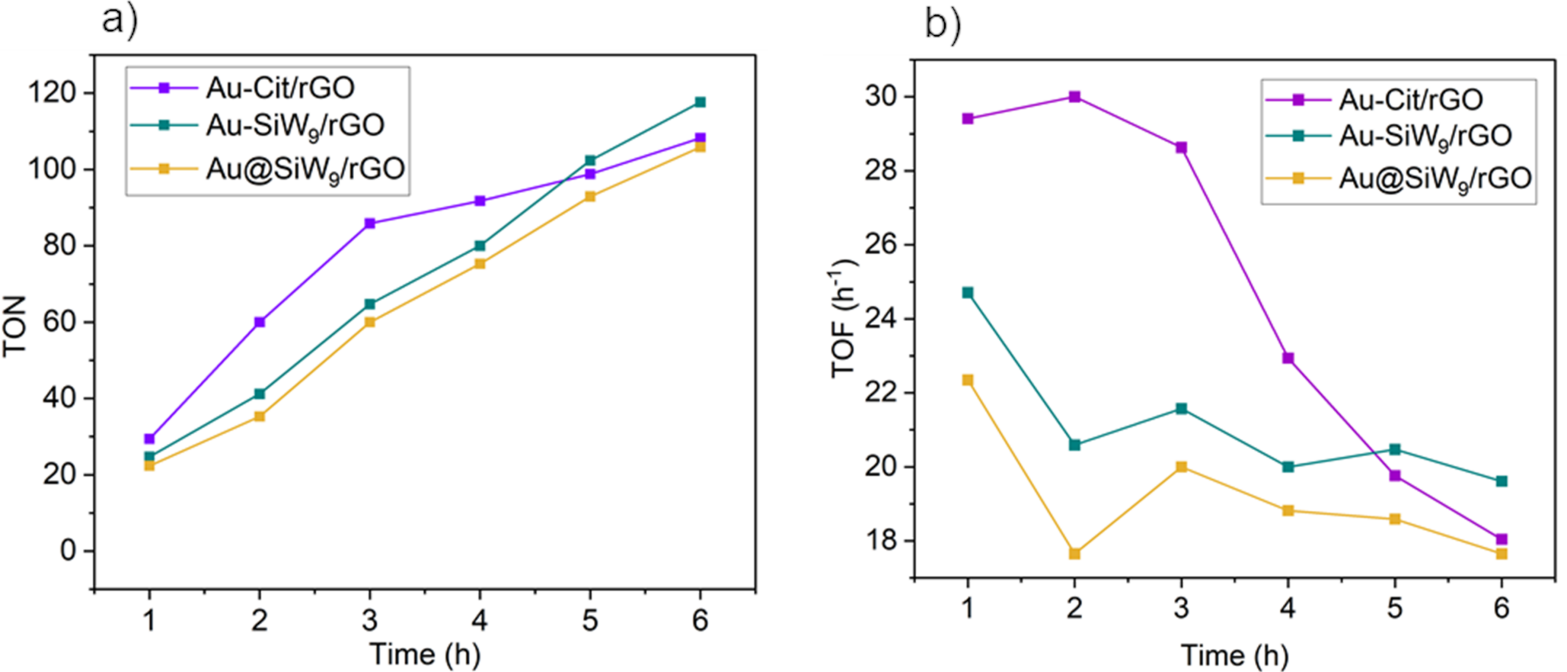
(a) Turnover number (TON) and turnover frequency (TOF) of the rGO-based catalysts measured in one hour intervals of the reaction (see Supporting Information File 1, Table S7 for the data).

The amount of supported gold in the composite was calculated by assuming quantitative conversion of the gold precursor to nanoparticles as well as quantitative uptake of the AuNPs (cf. Figure 5a and Figure 7a) onto the carbon support material. The progress of the reaction was followed by GC for the rGO catalyst composites (Figure 12a), and the reaction was stopped when 100% conversion was reached after 6 or 7 h for the most active systems. Also, for the less active, usually CB-based catalysts, the reaction was stopped after 8 h, and conversion and yield were determined.

All catalysts are 100% selective in the formation of *N*-methyl-4-piperidone to *N*-methyl-2,3-dihydropyridin-4(1*H*)-one; there are no side products. In the cases of less than 100% conversion, unreacted starting material accounted for the difference. Yields of less than 100% were due to the adsorption of product in the pores of the carbon materials. For the entries in Table 1 with less than 100% conversion (entry 3, 6, 7, and 9) extending the reaction time up to 10 or 15 h led only to a slight increase in conversion, except for the Au@SiW_9_/AC catalyst in entry 7, which reached 100% conversion after an additional 7 h (i.e., 15 h in total) (see the conversion data in Supporting Information File 1, Table S3).

To further validate the catalytic performance, we also investigated the ODH of *N*-ethyl- and *N*-benzyl-4-piperidone using the most active Au-SiW_9_/rGO composite catalyst. In these cases, the corresponding *N*-alkyl-2,3-dihydropyridin-4(1*H*)-one products were obtained with 100% conversion and selectivity (Table 2).

**Table 2:** Catalytic results for the *N*-ethyl- and *N*-benzyl-4-piperidone substrates.^a^

Entry	Substrate	Product	Catalyst (mg)^b^	Au/substrate^c^	Time (h)	Conversion (%)^d^	Selectivity (%)^e^

1	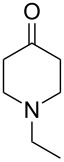	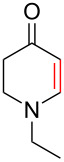	10	0.0085	7	100	100
2	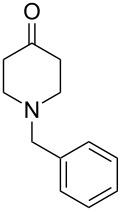	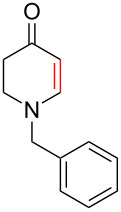	70	0.060	24	25	89
3			50	0.043	24	67	97
4			30	0.026	24	97	98
5			10	0.0085	18	100	100

^a^Reaction conditions: 0.25 mmol of N-substituted piperidones; 2 mL of water, air 1.013 bar, temperature 90 °C. Further catalytic results and ^1^H NMR characterization are provided in Supporting Information File 1 (Figures S12–S15). ^b^Amount of applied Au-SiW_9_/rGO with 4.1 wt % of Au in the catalysis. ^c^mol Au/mol piperidone; ^d^Conversion was determined from the molar concentration of the starting material before the reaction minus the starting material after the reaction divided by the concentration of the starting material before (×100%). ^e^Selectivity was determined as the molar amount of the desired product formed divided by the total molar amount of product formed, including by-products (×100%).

All three *N*-alkyl-4-piperidone substrates were successfully converted to their corresponding *N*-alkyl-2,3-dihydropyridin-4(1*H*)-one products using the Au–SiW_9_/rGO catalyst. However, the reactions required higher temperatures (90 °C) and longer reaction times (7 to 24 h) for *N*-ethyl- and *N*-benzyl- compared to *N*-methyl-4-piperidone. This behavior is consistent with the increased steric bulk, which make the α-hydrogen abstraction step more difficult. The observed catalytic trends further confirm the robustness and adaptability of the Au/rGO catalysts for ODH of structurally related β-N-substituted ketones. The corresponding catalytic data and product characterization have been included in Supporting Information File 1.

As shown in Table 1, catalysts with CB as support demonstrated lower conversion and yields, while AuNPs supported on AC and rGO achieved nearly complete or complete conversion of the reactant over time. However, AC was slightly less effective than rGO as a support because it required a longer reaction time to reach full conversion (8 h vs 7 or 6 h). This difference may be due to the surface chemistry and the predominantly microporous structure of activated carbon, which impedes efficient mass transport and diffusion of reactants. For Au@SiW_9_-AC, only 85% conversion was reached after 8 h, whereas the rGO equivalent achieved 100%. Conversely, the textural and morphological properties of rGO (Figure 2), characterized by increased mesoporosity (Figure 3), create a more favorable environment for catalyst dispersion, thereby enhancing reaction kinetics. This is likely due to the residual oxygen content of rGO (Supporting Information File 1, Figure S6), which enables the effective anchoring of AuNPs and results in higher catalytic efficiency. In addition, the AC-based composites tend to retain the product within their microporous structure even after multiple washings, resulting in a lower yield of the desired product compared to the rGO composites, despite the same 100% conversion. In contrast, all catalysts supported on rGO (entries 2, 5, and 8 in Table 2) consistently exhibited a high yield of 98%. This enhanced performance can be attributed to the structural characteristics of rGO, which facilitate more efficient mass transfer and enable complete release of the product after the reaction and subsequent washing steps. Compared to AC and CB, the thin, wrinkled, and sheet-like layers and lower microporosity of rGO (Figure 2 and Figure S1, Supporting Information File 1) minimize product entrapment, thereby enhancing overall catalytic efficiency. These textural properties make rGO the most effective support for AuNP catalysts.

For the AC- and CB-based composites, a higher catalytic activity of nanoparticles synthesized by RD (entries 1–6, Table 1) compared to those formed by DP (entries 7–9, Table 1) can be attributed to the AuNP dispersion and aggregation. In RD synthesis (Figure 1a,b), preformed AuNPs are adsorbed onto the carbon surface in a controlled manner, ensuring uniform dispersion (Figure 5 and Figure 7) and consequently maximal exposure to reactants. This enhances catalytic efficiency by providing greater access to active sites. Conversely, the DP synthesis (Figure 1c) results usually in less uniform particles due to uncontrolled nucleation and growth, which reduces the active surface area. Moreover, AuNPs become partially embedded within the carbon matrix, reducing their access and availability for catalytic reactions [46]. Consequently, the composites prepared by RD typically exhibit superior catalytic performance compared to their DP-derived counterparts.

To enable a meaningful comparison of the catalysts’ activity over time, turnover number (TON) and turnover frequency (TOF) values were analyzed after each hour from one to six hours (Figure 11a and Supporting Information File 1, Table S7). The catalytic performance over time underscores the long-term stability of Au-SiW_9_/rGO. It not only achieved the highest TON (118) among the three catalysts but also maintained a steady increase in TON and only a slight decrease in TOF after two hours, reflecting its ability to continuously convert substrate over time with minimal deactivation. In contrast, Au-Cit/rGO, despite its initially high activity, experienced a significant decline, with TOF dropping to 18 h^−1^ and TON reaching 108, indicating a notable reduction in catalytic efficiency, despite the much higher gold loading (Table 2). Au@SiW_9_/rGO exhibited the lowest values after 6 h (TON = 106, TOF = 18 h^−1^), confirming its comparatively lower overall catalytic performance. This trend corroborates the earlier observation that Au-Cit/rGO is more effective for short reaction times, whereas Au-SiW_9_/rGO proves to be the most robust and efficient for prolonged catalytic processes. The catalyst Au@SiW_9_ also appears to be suitable for long-term reactions. Although it exhibits a slower reaction rate due to the preparation method as discussed earlier, it maintains a more consistent catalytic activity over time than Au-Cit/rGO.

**Figure 13:**
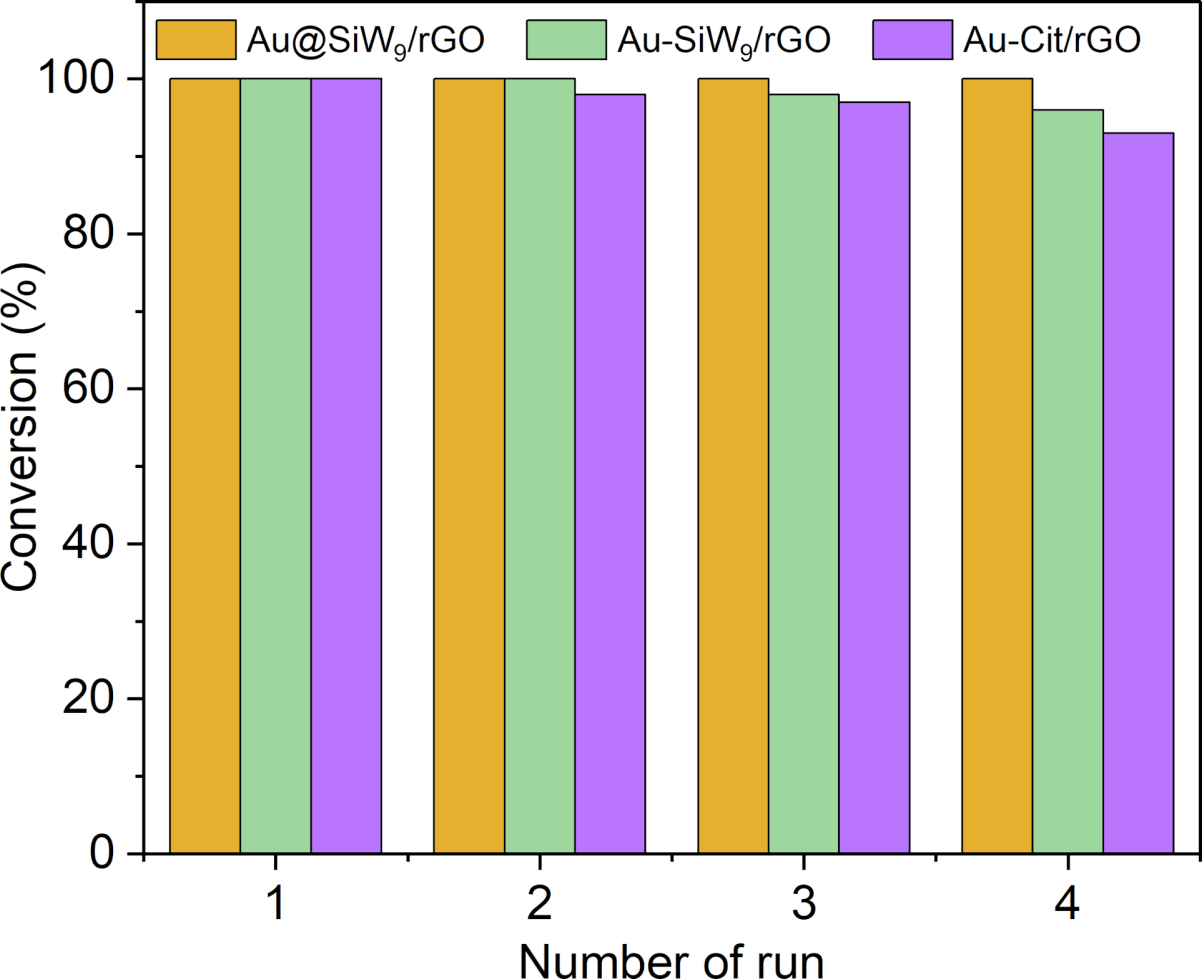
Catalytic stability test for the rGO-based catalysts.

An important aspect of the AuNP heterogenization on a carbon support is the separation, recycling, and reuse of the catalyst, which also concerns the stability of the catalyst. This was tested for the most active rGO-based catalysts over four runs. The catalysts were separated by filtration using standard filter paper, then thoroughly washed and dried before being reused in subsequent reaction cycles. In fact, catalytic stability tests after the four reaction runs (Figure 13) show that for the Au-Cit catalyst, the conversion within the fixed time of 7 h dropped from 100% to 89%, which was more than for the Au-SiW_9_ catalysts (6 h), which dropped from 100% to only 94%. The Au-Cit and Au-SiW_9_ catalysts were prepared by the RD method. The Au@SiW_9_/rGO catalyst, which was obtained by the DP method, retains its full catalytic activity with 100% conversion in 7 h even after four cycles (Figure 13). These results highlight the effectiveness of the POM SiW_9_ in stabilizing the nanoparticles, thereby preserving their catalytic activity over recycling and extended use.

Thus, the AuNPs on rGO synthesized by the RD method appear to be less stable and become less catalytically active over time than those formed via DP. This may be due to the metal–support interaction in RD, where the citrate or SiW_9_ POM-coated AuNPs adhere to rGO mainly through electrostatic forces rather than direct AuNP-rGO chemical bonding. As a result, these nanoparticles are more susceptible to detachment and aggregation over time, especially under catalytic conditions. In contrast, DP leads to in situ nucleation and growth of AuNPs directly on the rGO surface, with the possibility of forming direct metal–oxygen bonds to rGO that enhance particle anchoring. Therefore, while RD-synthesized AuNPs may offer better dispersion and catalytic accessibility, they are inherently less stable compared to those formed by DP due to weaker metal–support interactions and a tendency for leaching over several cycles.

Beyond the structural advantages of mentioned earlier, rGO also provides a unique electronic environment that further enhances catalytic activity. The superior catalytic performance of AuNPs–rGO compared to Au supported on CB or AC therefore also stems from the synergistic interfacial C–O–Au arrangements, which act as bifunctional active sites for both, electron transfer and oxygen activation.

**Figure 14:**
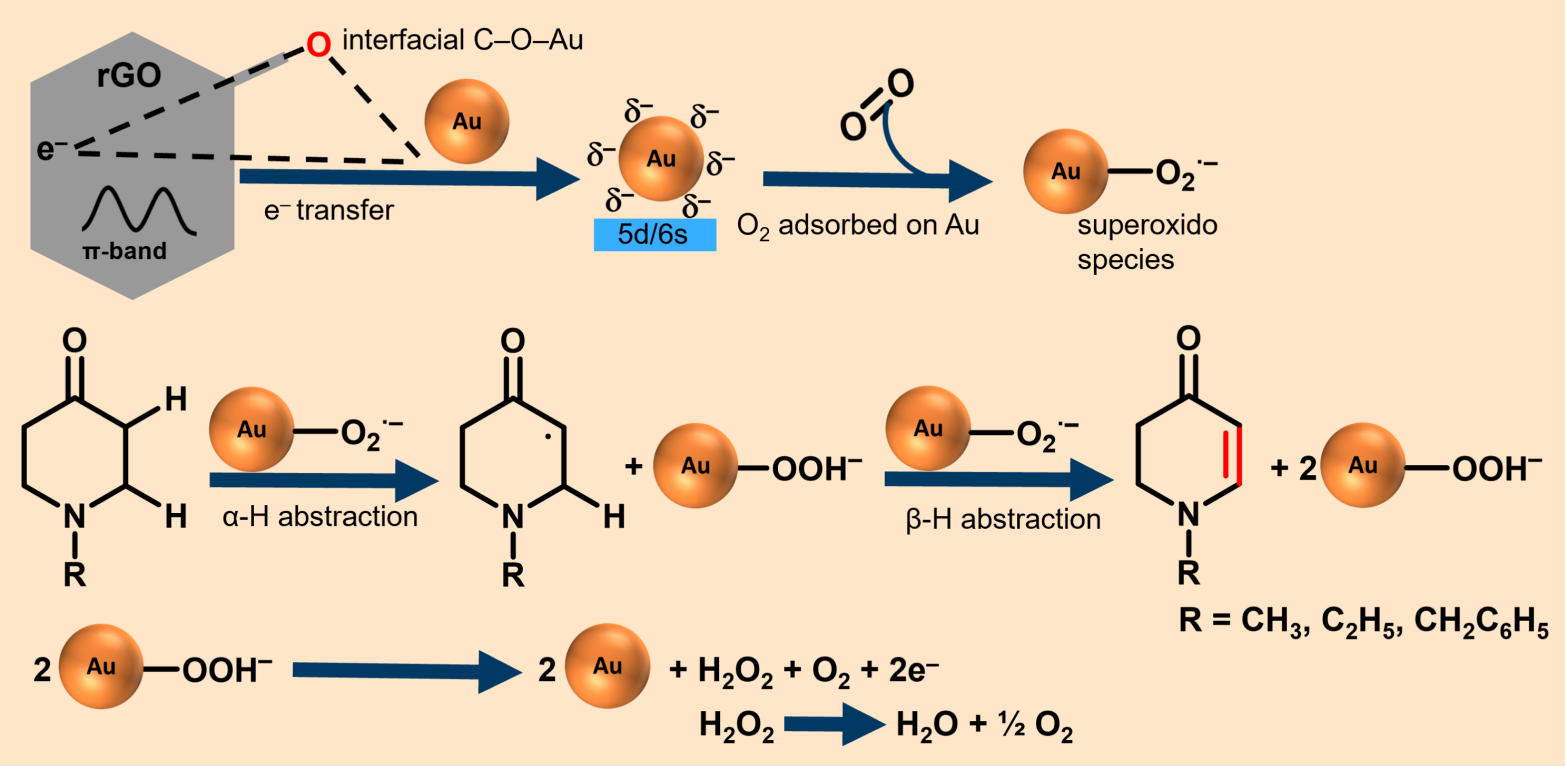
Possible reaction pathways for AuNPs/rGO-catalyzed aerobic oxidative α,β-dehydrogenation.

The oxygenated functional groups of rGO (–OH, C=O, –CO) (Supporting Information File 1, Figure S6) anchor and electronically couple gold nanoparticles to the π-conjugated sp^2^ carbon network. The π-conjugated domains of rGO, acting as an electron reservoir, raise the local Fermi level [47] and supply electron density directly into the interfacial Au 5d/6s orbitals via both the C–O–Au bridge and direct π–metal coupling [48]. This Fermi-level alignment enables rGO to inject electrons into Au even without permanent O-bridging, rendering the interfacial Au more electron-rich (Figure 14). XPS analysis supports this electronic interaction, showing a negative shift of the Au 4f binding energy (Figure 11a).

Upon O_2_ adsorption, electron back-donation from Au into the O_2_ π* orbitals generates activated Au–O_2_˙^−^ (superoxido) or Au–O_2_^2−^ (peroxido) species capable of abstracting the α-H atom from the N-substituted piperidone to initiate the α,β-dehydrogenation pathway [49–50]. In this study, the Au–O_2_˙^−^ species predominate, since the metallic AuNPs (3–13 nm), assisted by electron donation from rGO’s π-conjugated and oxygenated domains, provide sufficient but not excessive charge transfer to partially activate O_2_, yet without full reduction to the peroxido form. This balance maintains high selectivity while preventing overoxidation or uncontrolled H_2_O_2_ formation. The resulting Au–OOH^−^ (hydroperoxido) intermediate subsequently decomposes to oxygen and H_2_O_2_, which is rapidly converted to H_2_O and O_2_ [51]. This decomposition regenerates the active Au–O_2_˙^−^ species allowing the catalytic cycle to continue. Consequently, rGO not only stabilizes Au nanoparticles but also sustains dynamic charge transfer (rGO→Au→O_2_), preserving the redox flexibility of Au sites throughout the catalytic cycle.

A very important point to consider when using carbon supports in catalysis is the gold loading in the support. Compared to AC and CB, rGO exhibits a lower bulk density due to its high porosity than the other carbon materials AC and CB, as illustrated in Supporting Information File 1, Figure S4. When comparing the two reduction methods, hot reduction with NaCit and cold reduction with POM (SiW_9_), important differences arise in nanoparticle formation. NaCit is a mild reducing agent that reduces Au^3+^ slowly, leading to gradual nucleation. As a result, fewer gold nuclei form, and each nucleus has time to grow into a larger particle. In contrast, NaBH_4_ is a strong reducing agent that rapidly reduces Au^3+^ under cold conditions, triggering a burst of nucleation. This produces many small gold nuclei almost instantly, leaving little precursor available for further growth. The outcome is a high number of small AuNPs with greater surface area and a higher number of catalytically active sites. Beyond the improved stability of AuNP-based POMs, attributable to the steric bulk of the POMs, this fundamental difference in synthesis also helps to explain why the Au-SiW_9_/AC and Au-SiW_9_/rGO catalysts, despite having more than two times lower gold loading than the Au-Cit/AC and Au-Cit/rGO catalysts (entries 4 and 5 vs 1 and 2, Table 2, see also Table S6), still exhibit comparable catalytic activity.

The Au-SiW_9_/AC catalyst, originally synthesized by Xia et al. [13], was reproduced in this study with a key difference of gold loading. Xia et al. used 3.17 wt % Au, corresponding to 0.025 mol Au/mol substrate ratio in the oxidation reaction. In this study, we used only 0.42 wt % Au in the composite giving 0.0085 mol Au/mol substrate in the catalysis, yet achieved better conversion and higher yield for the same target reaction. Xia et al. reported a conversion of 92% and a yield of 86% as their best result. This clearly highlights that the catalytic activity of gold is not solely dependent on the amount of gold, but rather on the dispersion of gold nanoparticles, which increases the number of accessible active sites and reduces the need for excessive gold loading. In fact, increasing gold content can be detrimental, as it may lead to particle aggregation, thereby reducing the surface area and number of active sites. Thus, the superior performance of the lowly loaded Au-SiW_9_/AC catalyst synthesized in this study compared to the higher-loaded Au-SiW_9_/C catalyst reported by Xia et al. demonstrates the critical role of nanoparticle dispersion and size control in achieving efficient catalytic performance.

Regarding the explanation about the lower metal loading, not only does the catalytic reaction proceed with a lower gold loading, a lower metal loading in the Au/rGO catalyst actually enhances the catalytic activity in the ODH reaction. This effect has been demonstrated here using the most challenging (that is, least easily oxidized) substrate *N*-benzyl-4-piperidone (entries 2–5, Table 2). Catalytic tests were performed with 10, 30, 50, and 70 mg of catalyst. The results demonstrate a pronounced inverse relationship between catalyst amount and catalytic performance. As the catalyst mass decreases from 70 to 10 mg, both conversion and selectivity increase substantially. At the highest catalyst loading (70 mg, 0.060 mol Au/mol substrate), the reaction exhibits the lowest conversion (25%) and least selectivity (89%) after 24 h of reaction time. In contrast, the lowest catalyst loading (10 mg) achieves full conversion with 100% selectivity in a shorter reaction time (18 hours). These findings indicate that excess catalyst negatively affects the ODH reaction, likely by promoting undesired secondary pathways or increasing overoxidation. Optimal activity and selectivity are therefore achieved only at lower catalyst amounts, where the reaction proceeds more cleanly and efficiently. Supporting GC spectra for the different catalyst loadings are included in Supporting Information File 1 (Figure S9), providing additional evidence for the results observed in Table 2.

Two complementary factors can explain the effect of catalyst amount on the ODH reaction. First, higher catalyst loading can introduce mass transfer and diffusion limitations. Increasing the amount of catalyst raises the suspension density, which restricts the diffusion of both the substrate and dissolved O_2_ to the inner catalytic surfaces. Because the ODH reaction depends on molecular oxygen as the oxidant, such limitations lead to both external and internal diffusion constraints. Consequently, the apparent reaction rate decreases – not due to a loss of intrinsic catalytic activity, but because mass transfer fails to supply O_2_ rapidly enough to sustain the reaction at its potential rate. This interpretation is consistent with recent findings showing that increasing the amount or granularity of supported-metal catalysts can induce both external and internal mass transport limitations, ultimately suppressing the observed reaction rate under otherwise kinetically favorable conditions [52].

Second, excessive catalyst loading can promote recombination or quenching of reactive oxygen species generated on the Au/rGO surface. As demonstrated in the proposed ODH mechanism (Figure 14), Au/rGO activates O_2_ to form reactive O_2_˙^−^ species. At higher catalyst concentrations, the local density of these species increases, enhancing the probability of bimolecular recombination or radical quenching (e.g., O_2_˙^−^ + O_2_˙^−^ → O_2_ + O_2_^2−^ or 2HO_2_˙ → H_2_O_2_ + O_2_). This undesired recombination reduces the availability of active oxygen intermediates required for α,β-hydrogen abstraction, thereby decreasing the overall catalytic efficiency.

Minor by-products that have been observed for oxidative dehydrogenation/oxidation of *N*-methyl piperidone-type substrates include *N*-oxides (oxidation at the tertiary N), oxidative N-demethylation products, and overoxidized carbonyls (α-diketones and ultimately carboxylic acids); under strongly radical or overoxidative conditions, one can also observe fragmentation/ring-opening products. This behavior is documented in mechanistic studies of ODH of *N*-methyl-4-piperidone [50] and in more general literature on the oxidation for tertiary amines and cyclic ketones. This trend is clearly illustrated in our own experiments on the ODH of *N*-benzyl-4-piperidone to *N*-benzyl-2,3-dihydropyridin-4(1*H*)-one (Table 2). At high catalyst loading (70 mg, 0.06 mol Au/mol piperidone), we observe lower conversion (25%) and reduced selectivity (89%), consistent with the formation of small amounts of secondary oxidation products. In contrast, reducing the catalyst mass progressively suppresses these undesired pathways. At 10 mg (0.0085 mol Au/mol piperidone), the reaction proceeds cleanly with 100% selectivity and full conversion, and no by-products are detected by GC (Supporting Information File 1, Figure S8). These results demonstrate that excess catalyst can indeed promote minor by-product formation, whereas under the optimized low-catalyst conditions used in the present study, such pathways are efficiently suppressed, explaining the observed 100% selectivity.

**Figure 15:**
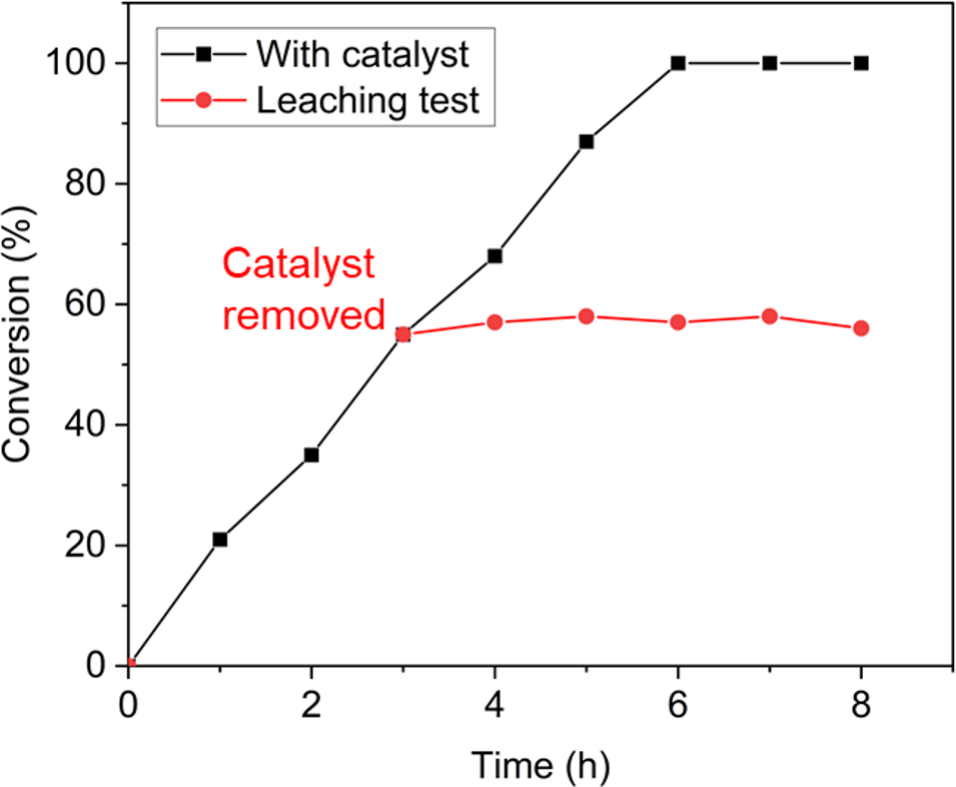
Leaching test of the Au–SiW_9_/rGO catalyst.

Because the Au-SiW_9_/rGO composite exhibited the most promising activity among the tested catalysts, its structural robustness was examined through leaching experiments and TEM imaging after successive reaction cycles. To confirm the solid-state (heterogeneous) nature of the catalyst, a leaching (hot filtration) test was performed. The reaction was conducted for 3 h under standard conditions, after which the catalyst was removed by hot filtration, and the filtrate was further stirred under identical conditions. Only a minor increase in conversion was observed after catalyst removal, indicating that the catalytic activity almost completely stopped in the absence of the solid catalyst (Figure 15). Moreover, AAS analysis of the filtrate detected only trace amounts of Au (around 0.06 mg·L^−1^), confirming negligible leaching. This may be due to a few small AuNPs, which were not retained by the used MN 615 1/4 cellulose filter paper with 4.0–12.0 µm pore sizes [53]. These results unambiguously demonstrate that the reaction proceeds via a truly heterogeneous catalytic mechanism, with Au nanoparticles remaining immobilized on the rGO support throughout the reaction runs.

**Figure 16:**
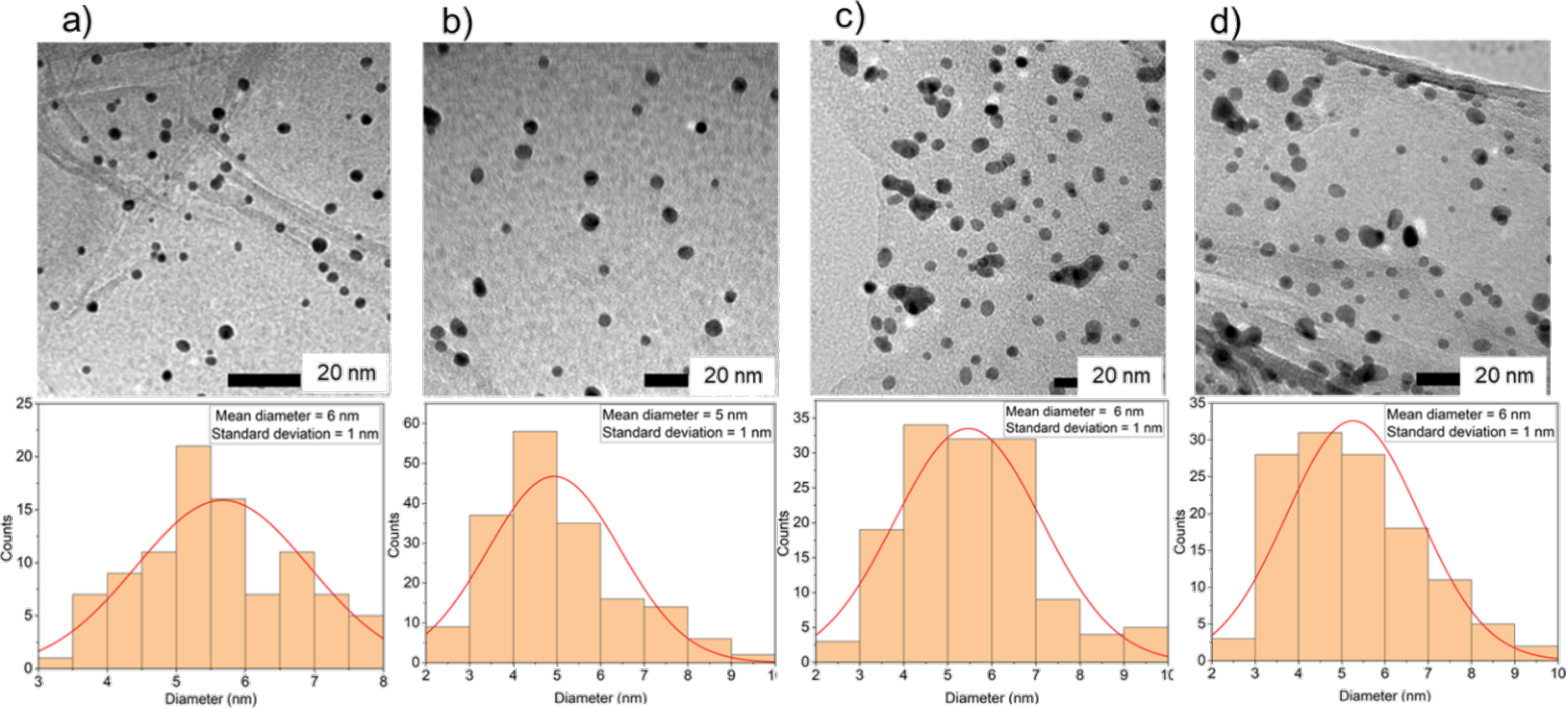
TEM analysis of the Au–SiW_9_/rGO catalyst over three consecutive catalytic runs: (a) fresh catalyst, (b) after the first run, (c) after the second run, and (d) after the third run.

Post-mortem TEM analysis shows that the Au nanoparticles remain well dispersed even after the third catalytic run (Figure 16). A slight aggregation appears from the second run, but the distribution histogram indicates only a small increase in particle size, with the average diameter essentially unchanged at approximately 6 nm. This confirms that the nanoparticles remain stable during repeated use, which is consistent with the stability tests presented in Figure 13, where the Au–SiW_9_/rGO catalyst maintained its catalytic performance over three consecutive runs, confirming both its structural stability and recyclability.

Although the selective α,β-dehydrogenation of β-N-substituted saturated ketones is a reaction of considerable importance in both chemistry and medicine, it is noteworthy that relatively few studies have examined the catalytic activity of supported metal nanoparticles for this type of transformation, particularly for the oxidative dehydrogenation of *N*-alkyl-4-piperidone to the corresponding *N*-alkyl-2,3-dihydropyridin-4(1*H*)-one. Table 3 compares the *N*-methyl-4-piperidone substrate in this work with the best results reported in the literature using supported metals. Notably, compared to literature reports, quantitative conversion and yield have been achieved in shorter reaction times and with lower gold loadings (Table 3) using the novel catalyst synthesized in this work.

**Table 3:** Comparison of catalytic studies on the oxidative dehydrogenation of 1 *N*-methyl-4-piperidone to *N*-methyl-2,3-dihydropyridin-4(1*H*)-one with metal nanoparticles.

Catalysts	Solvent	Temperature (°C)	Time (h)	Conversion (%)	TON^a^	TOF^b^ (h^–1^)	Yield (%)	Ref.

Au-POM/AC	water	50	8	92	37	5	86	[13]
Au/OMS-2^c^	water	50	2	94	26	13	90	[54]
Pd/Au/CeO_2_	dimethylacetamide	70	1	/	14	14	79	[55]
Au-SiW_9_/rGO	water	60	6	100	118	20	98	this work
Au-Cit/rGO	water	60	7	100	47	8	98	this work
Au@SiW_9_/rGO	water	60	7	100	118	15	97	this work

^a^TON = moles of product formed/moles of metal (of the catalyst); ^b^TOF = TON/time; ^c^OMS-2 = manganese oxide octahedral molecular sieve.

## Conclusion

This study demonstrates that the catalytic performance of AuNP-based catalysts is strongly influenced by the carbon support, synthesis method, and stabilizing ligand. Among the supports evaluated, reduced graphene oxide (rGO) provides the most favorable combination of mesoporosity and surface functionality, enabling superior nanoparticle stabilization and enhanced reactant diffusion. The Au/rGO catalysts synthesized via the reduction–precipitation method and stabilized with SiW_9_ polyoxometallate show the highest efficiency in the oxidative dehydrogenation (ODH) of *N*-methyl-4-piperidone. The superior activity of these catalysts arises from electron transfer from rGO to Au via C–O–Au interfacial sites. XPS confirms the formation of the active Au–SiW_9_/rGO structure, and TEM demonstrates structural stability over multiple runs. Leaching tests verify the solid-state heterogeneous nature of the catalyst. Importantly, the catalyst maintains high activity even at low Au loading and efficiently promotes ODH of additional substrates, including *N*-ethyl-4-piperidone and *N*-benzyl-4-piperidone. Compared to previously reported systems, the Au/rGO catalysts achieve higher yields and shorter reaction times, highlighting rGO as a promising support for designing efficient, cost-effective, and scalable heterogeneous catalysts. These findings identify mesoporosity and electron-rich interfaces as key factors driving enhanced catalytic performance and provide a framework for future applications in α,β-dehydrogenation of β-N-substituted saturated ketones.

## Experimental

### Catalyst’s preparation

All chemicals used in this study were of analytical grade and were used as received without further purification: acetone (≥99%, Sigma-Aldrich), potassium tetrachloridoaurate(III) (≥99%, BLD PHARMATECH GmbH), sodium citrate dihydrate (≥99%, VWR Chemicals), sodium borohydride (≥96.0%, Merck KGaA), *N*-methyl-or 1-methyl-4-piperidone (≥99%, BLD PHARMATECH GmbH), sodium metasilicate nonahydrate (≥98%, Sigma-Aldrich), sodium tungstate (≥98%, Sigma-Aldrich), hydrochloric acid (37%, for analysis EMPARTA^®^ ACS, Merck), activated charcoal, DARCO^®^, −100 mesh particle form, nanostructured powder (Cabot Corporation), and sodium carbonate anhydrous (≥99.5%, Sigma-Aldrich).

Reduced graphene oxide (rGO) was synthesized through a two-step process involving oxidation followed by thermal reduction, starting from natural graphite (type KFL 99.5, supplied by AMG Mining AG, formerly Kropfmühl AG, Passau, Germany). The oxidation step was carried out using the method developed by Hummers and Offeman [56], and the thermal reduction was performed at 750 °C [57].

### Preparation of Au-Cit/AC, Au-Cit/rGO, and Au-Cit/CB by RD method

In a 500 mL Erlenmeyer flask, 20 mg (53 μmol) KAuCl_4_ were dissolved in 200 mL of water and brought to its boiling point at 100 °C on a magnetic stirring plate with a heating function while stirring continuously at 350 rpm. Once the solution reached the boiling point, 93 mg (319 μmol) of NaCit was added, causing the initially yellow solution to immediately become colorless, violet, and bright ruby red, indicating the formation of AuNPs [58]. The different carbon supports (AC: 1000 mg, rGO: 100 mg, CB: 500 mg) were then rapidly added (each in different batches of colloidal AuNPs) under vigorous stirring for another 15 min to ensure the complete deposition of AuNPs on the different carbon supports. The resulting suspension was then cooled to room temperature, filtered, and thoroughly washed several times with deionized water to remove the excess of sodium citrate. The retained solids were dried in a vacuum oven at 60 °C for 24 h and stored in a sealed glass for further utilizations.

#### Preparation of Au-SiW_9_/AC, Au-SiW_9_/rGO, and Au-SiW_9_/CB by RD method

The synthesis of colloidal AuNPs coated with SiW_9_ was based on a method reported by Xia and coworkers [13]. In a typical procedure, 50 mg of SiW_9_ was dissolved in 55 mL of water and placed in an ice bath (≈1 °C) to prevent isomerization of the polyoxometalate (POM). A 0.1 mol·L^−1^ aqueous solution of KAuCl_4_ (prepared by dissolving 5.7 mg of KAuCl_4_ in 150 μL of water) was then added to the SiW_9_ solution under stirring and allowed to react for 60 min. Subsequently, an ice-cold 0.03 mol·L^−1^ NaBH_4_ solution (5.6 mg of NaBH_4_ dissolved in 5 mL of water) was added dropwise under continuous stirring. Upon complete addition of NaBH_4_, the solution turned brown-orange, indicating the successful formation of AuNPs. Immediately after the NaBH_4_ addition, different carbon supports (AC: 700 mg, rGO: 70 mg, CB: 350 mg) were separately introduced into different batches of the colloidal AuNPs solution under vigorous stirring. The mixtures were stirred for an additional 30 min to ensure the complete deposition of AuNPs onto the carbon supports. The resulting suspensions were then filtered and thoroughly washed with deionized water to remove excess SiW_9_. The retained solids were dried in a vacuum oven at 60 °C for 24 h and stored in sealed glass containers for further use.

#### Preparation of Au@SiW_9_/AC, Au@SiW_9_/rGO, and Au@SiW_9_/CB by DP method

This synthesis followed the same protocol as described above using the RD method with SiW_9_, with the only difference being the order of reagent addition. After mixing the polyoxometalate (SiW_9_) and the gold precursor solution under the same conditions for 60 min, different carbon supports (AC: 700 mg, rGO: 70 mg, CB: 350 mg) were separately introduced into different batches of the mixture. The stirring was continued for an additional 60 min before the dropwise addition of NaBH_4_ under continuous stirring in each batch. After the reaction, the resulting suspension was cooled to room temperature, filtered, and thoroughly washed several times with deionized water to remove excess sodium citrate. The retained solids were then dried in a vacuum oven at 60 °C for 24 h and stored for further use.

#### Leaching test

The catalytic reaction was initially performed under standard reaction conditions for 3 h in the presence of the solid catalyst. After completion of this period, the catalyst was removed by hot filtration using a MN 615 1/4 cellulose filter paper (125 mm diameter) with 4.0–12.0 µm pore sizes to ensure complete separation of the solid from the reaction mixture. The resulting filtrate then underwent further reaction under identical conditions for an additional 8 h to assess the contribution of any leached active species. Aliquots of the filtrate (typically 0.5–1.0 mL) were withdrawn at hourly intervals starting from the fourth hour and analyzed to monitor the progression of the reaction and to detect any catalytic activity in the absence of the solid catalyst.

#### AAS measurement

The gold loading in the catalyst was determined by atomic absorption spectroscopy (AAS). For analysis, 5.0 mg of the Au-SiW_9_/rGO material was digested in a microwave digestion system using 3 mL of concentrated HNO_3_ (70%). After digestion, the solution was diluted to a final volume of 50 mL with 5% HCl. The resulting solution was analyzed by AAS, giving an Au concentration of 4.1 mg·L^−1^. This corresponds to 0.205 mg or 4.1 wt % of Au in the digested sample.

#### Catalytic procedure for the oxidation of *N*-methyl-4-piperidone

A total of 0.25 mmol of *N*-methyl-4-piperidone was dissolved in 2 mL of deionized water along with a specific amount of catalyst, which varied depending on the carbon support used in each reaction (see Supporting Information File 1 for details). The reaction was conducted in a glass tube under open-air conditions at 60 °C with vigorous stirring for 8 h. Upon completion, the reaction mixture was separated from the catalyst by filtration using filter paper. The catalyst was then washed multiple times with a precise volume of acetone. The collected filtrate was analyzed directly by gas chromatography.

**Recycling runs:** The reusability of the catalysts was evaluated under optimized reaction conditions over four consecutive cycles, as shown in Figure 13. After each run, the catalyst was recovered from the reaction mixture by simple filtration using filter paper. The recovered catalyst was then thoroughly washed with acetone (4 × 30 mL) and water (3 × 30 mL) to remove any adsorbed reactants or products, followed by drying in an oven at 60 °C for 24 h. The dried catalyst was subsequently reused in the next cycle under identical conditions without any additional catalyst being added.

#### Instrumental details

Gas chromatography (GC) analyses were carried out using a Shimadzu GC-2014 with an autoinjector AOC-20i equipped with an FID-2014 detector, FS-Supreme-5ms 25 mm × 0.25 mm × 0.25 µm column and HS-10 headspace sampler. Gas chromatography-mass spectrometry (GC-MS) spectra were performed using an Agilent technologies GC system equipped with an FID detector (7820A model), an auto-sampler (7693 model), and a mass-selective detector (MSD 5977E model), HP-5MS 30 m × 0.25 mm ID × 0.25 µm column. Proton nuclear magnetic resonance (^1^H NMR) was performed using a Bruker 300 MHz spectrometer. Scanning electron microscopy (SEM) were performed using a Jeol JSM-6510LV QSEM advanced electron microscope, operating at 20 kV with a LaB_6_ cathode. The instrument was equipped with a Bruker Xflash 410 silicon drift detector, enabling energy-dispersive X-ray (EDX) spectrometric analysis to determine the elemental composition of the materials. UV–vis spectroscopy spectral data were acquired using a VWR^®^ UV–visible spectrophotometer P9, covering wavelengths from 250 to 1100 nm. Dynamic light scattering (DLS) was carried out using a Malvern Nano S Zetasizer equipped with a helium–neon (HeNe) laser operating at 633 nm. Transmission electron microscopy (TEM) was performed using a JEOL JEM-2100Plus electron microscope operating at 200 kV, coupled with a Matataki Flash camera. With the Gatan Digital Micrograph software (version 3.61), the size of over 200 particles was determined regarding average diameter and size distribution. Atomic absorption spectroscopy (AAS) measurements were performed using a PerkinElmer PinAAcle 900T atomic absorption spectrometer. The instrument was operated in flame mode with an acetylene/air flame. Gold was measured at a wavelength of 242.80 nm using a slit width of 0.7 nm. Sample digestion prior to analysis was carried out with a CEM MARS 6 microwave digestion system. X-ray photoelectron spectroscopy (XPS) measurements were performed using an ULVAC-PHI VersaProbe II microfocused X-ray photoelectron spectrometer. Spectra were acquired with a polychromatic Al Kα X-ray source (1486.8 eV) and referenced to the C 1s peak at a binding energy of 284.8 eV. Data processing was carried out using the CasaXPS software (version 2.3.19PR1.0). Powder X-ray diffraction patterns were recorded using a Rigaku MiniFlex600 powder diffractometer in θ/2θ geometry (600 W, 40 kV, 15 mA). The measurements were carried out at ambient temperature using Cu Kα radiation (1.54182 Å) with the sample deposited on a rotating low-background silicon sample holder. N_2_ adsorption experiments were done on the BELSorp-max II (MicrotracBEL Corporation).

## Supporting Information

The Supporting Informations contains additional experimental details, pore size distribution analysis, BET measurements, powder X-ray diffraction (PXRD) measurement, TEM analysis, carbon support images, gas chromatography (GC), gas chromatography-mass spectrometry (GC-MS), ^1^H NMR spectroscopy spectra, gold loading, turnover number, and turnover frequency.

File 1 Additional experimental details and spectra.

## Data Availability

All data that supports the findings of this study is available in the published article and/or the supporting information of this article.
