# Supplementary material for: Gold nanoparticle-decorated reduced graphene oxide as a highly effective catalyst for the selective α,β-dehydrogenation of N-alkyl-4-piperidones
Source: Beilstein J Nanotechnol. 2026 Jan 30;17:218–38. doi: 10.3762/bjnano.17.15 (PMC12862619; doi:10.3762/bjnano.17.15)
Supplement: File 1 — Additional experimental details and spectra. [file Beilstein_J_Nanotechnol-17-218-s001.pdf]

BEILSTEIN JOURNAL OF NANOTECHNOLOGY

Supporting Information

for

Gold nanoparticle-decorated reduced graphene oxide as a highly effective catalyst for the selective α,β-dehydrogenation of *N*-alkyl-4-piperidones

Brenda Flore Kenyim, Mihir Tzalis, Marilyn Kaul, Robert Oestreich, Aysenur Limon, Chancellin Pecheu Nkepdep and Christoph Janiak

Beilstein J. Nanotechnol. **2026,** *17,* 218–238. doi:10.3762/bjnano.17.15

Additional experimental details and spectra

License and Terms: This is a supporting information file under the terms of the Creative Commons Attribution License (https://creativecommons.org/licenses/by/4.0). Please note that the reuse, redistribution and reproduction in particular requires that the author(s) and source are credited and that individual graphics may be subject to special legal provisions.
The license is subject to the *Beilstein Journal of Nanotechnology* terms and conditions: (https://www.beilstein-journals.org/bjnano/terms)

Additional experimental details

BET determination

Table S1: BET surface area and porosity data of the three carbon materials.

Sample	BET surface area ($m^2 \cdot g^{-1}$)[a]	Micropore volume ($cm^3 \cdot g^{-1}$)[b]	Total pore volume ($cm^3 \cdot g^{-1}$)[c]
AC	751	0.48	0.81
rGO	231	0.06	2.03
CB	167	0.07	0.57

Table S2: BET surface area and porosity data of rGO and its AuNP composites.

Sample	BET surface area ($m^2 \cdot g^{-1}$)[a]	Micropore volume ($cm^3 \cdot g^{-1}$)[b]	Total pore volume ($cm^3 \cdot g^{-1}$)[c]
rGO	231	0.06	2.03
Au-Cit/rGO	117	0.027	0.61
Au-SiW_9/rGO	106	0.026	0.60
Au@SiW_9/rGO	100	0.022	0.59

[a] Calculated BET surface area from N_2 adsorption at 77 K over a pressure range of the BET plot of P/P_0 = 0.01–0.07.

[b] Micropore volume from the NL-DFT method using the N_2 adsorption isotherm at 77 K at P/P_0 = 0.1 for pores with $d \leq 2$ nm (20 Å).

[c] Total pore volume from N_2 adsorption isotherm at 77 K at P/P_0 = 0.90 for pores smaller than 40 nm.

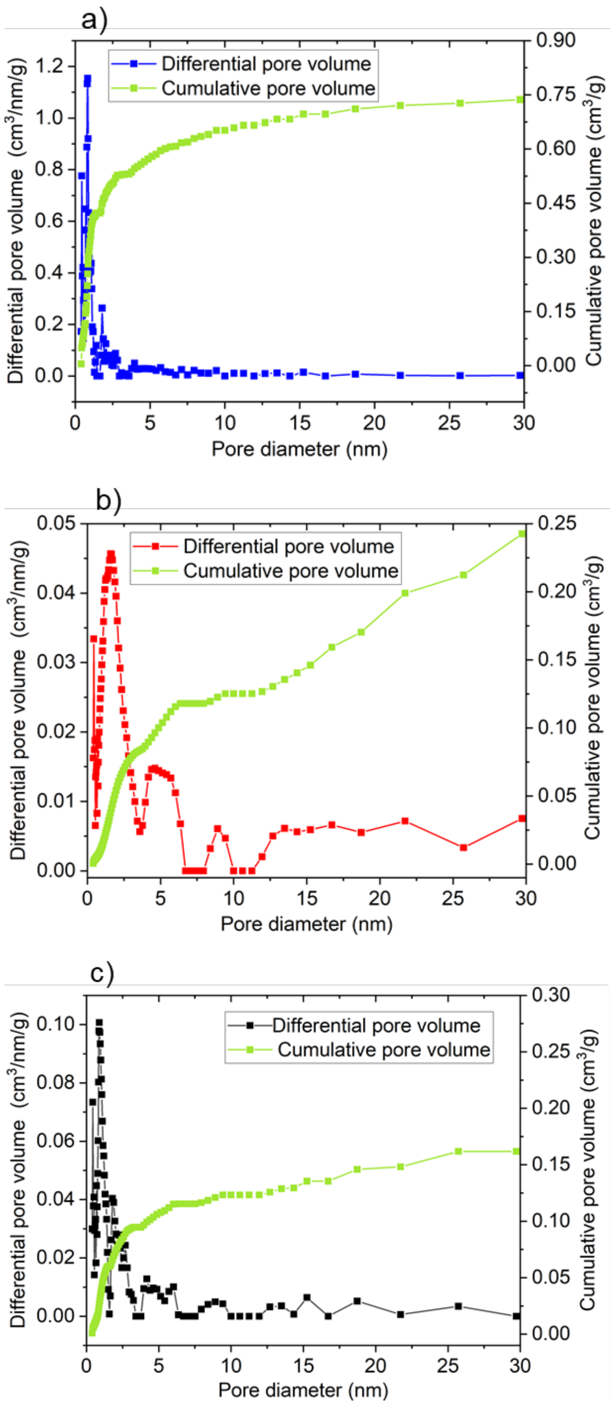

Figure S1: Pore size distributions curves of a) AC, b) rGO, c) CB.

TEM analysis

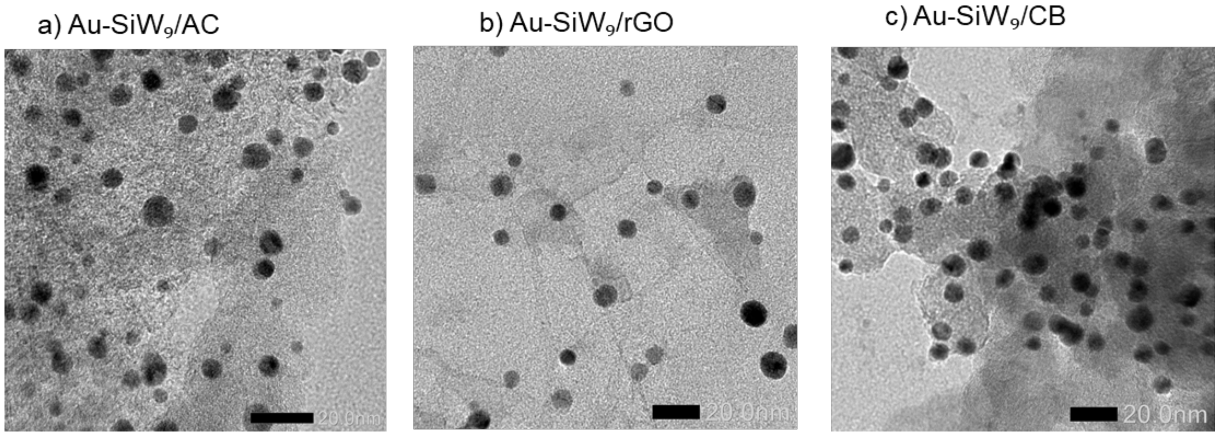

Figure S2: TEM images of a) Au-SiW_9/AC, b) Au-SiW_9/rGO, and c) Au-SiW_9/CB at higher resolution than in Figure 7. The scale bar is 20 nm.

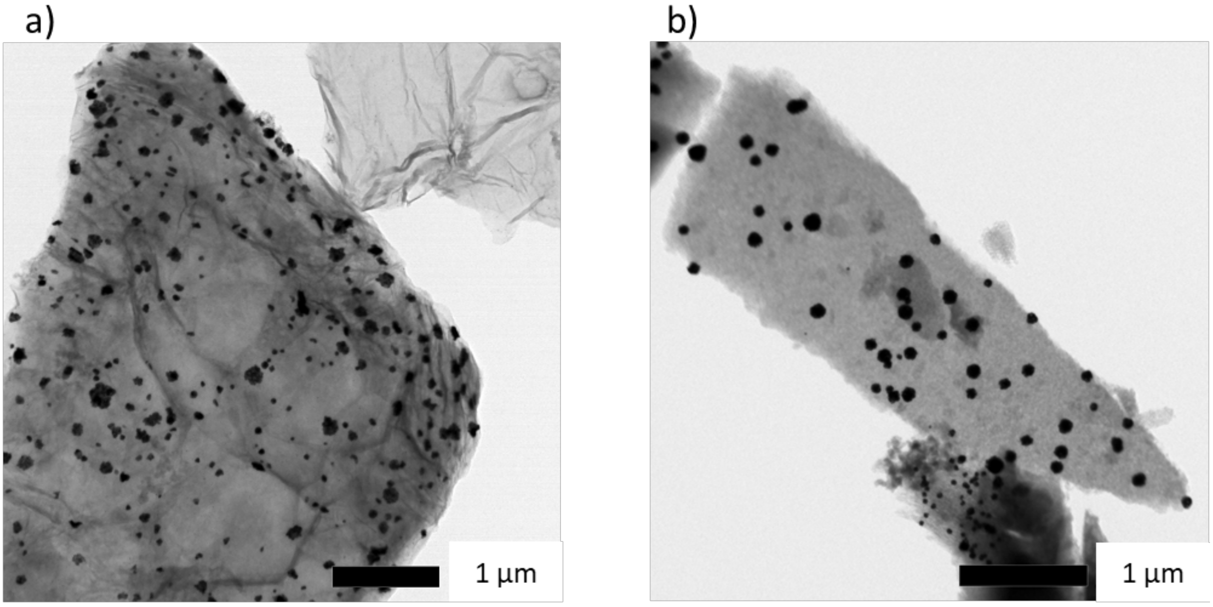

Figure S3: TEM images of aggregated particles by the deposition precipitation method of a) Au@SiW_9/rGO and b) Au@SiW_9/AC.

Carbon material images

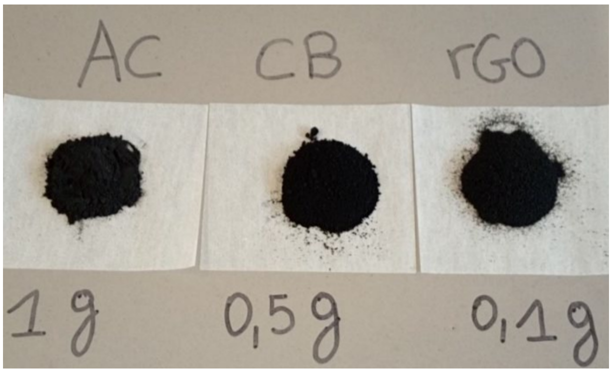

Figure S4: Illustrative images of the bulk density difference for similar volumes of the carbon supports AC, CB, and rGO.

Powder X-ray diffraction (PXRD)

The Powder X-ray Diffraction was recorded using the Rigaku MiniFlex600 powder diffractometer in θ/2θ geometry (600 W, 40 kV, 15 mA). The measurements were carried out at ambient temperature using Cu Kα radiation (1.54182 Å) with the sample deposited on a rotating low-background silicon sample holder.

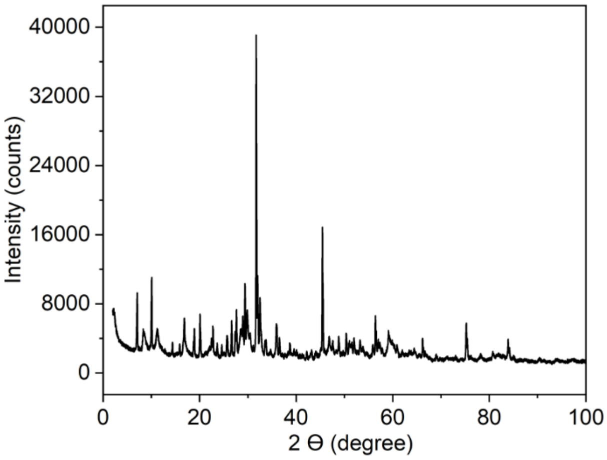

Figure S5: PXRD of POM SiW_9. To the best of our knowledge, no PXRD pattern of SiW_9 has been reported in the literature. Therefore, we are unable to provide a diffraction pattern for comparison or discuss characteristic peaks.

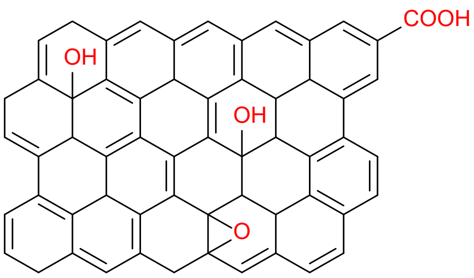

Figure S6: Schematic drawing of a section of a reduced graphene oxide (rGO) sheet with residual oxygen groups.

Table S3: Extended reaction time for the ODH of *N*-methyl-4-piperidone to *N*-methyl-2,3-dihydropyridin-4(1*H*)-one.

		Conversion after reaction time of		
Entry from Table 1	Catalyst	8 h	10 h	15 h
3	Au-Cit/CB	60%	63%	67%
6	Au-SiW_9/CB	42%	43%	45%
7	Au@SiW_9/AC	85%	92%	100%
9	Au@SiW_9/CB	23%	23%	25%

[a] Reaction conditions: 30 mg, 0.25 mmol of *N*-methyl-4-piperidone, 2 mL of water, open air 1.013 bar, temperature 60 °C. The catalyst amounts are as given in Table 1 in the main text.

Gas chromatography (GC)

GC was employed to analyze the reaction mixture, providing detailed information on the composition and progress of the reaction. The sample was prepared by transferring 1 mL of the reaction mixture dissolved in acetone into a GC vial. The analysis was performed using the parameters given in the following Table S4.

Table S4: Conditions for GC measurements.

Parameter	Value
Column	FS-Supreme-5ms 25 m; 0.25 mm; 0.25 µm
Carrier gas	H_2 3.0 mL/min
Injection	1 µL at 250 °C
Split flow	46.3 mL/min
Split ratio	30
Detection	FID
FID temperature	350 °C
Makeup gas	100 kPa (Nitrogen)
Gradient	0 min: 40 °C; 3 min: 40 °C; 30 °C/min to 240 °C; 6 min at 240 °C

a)

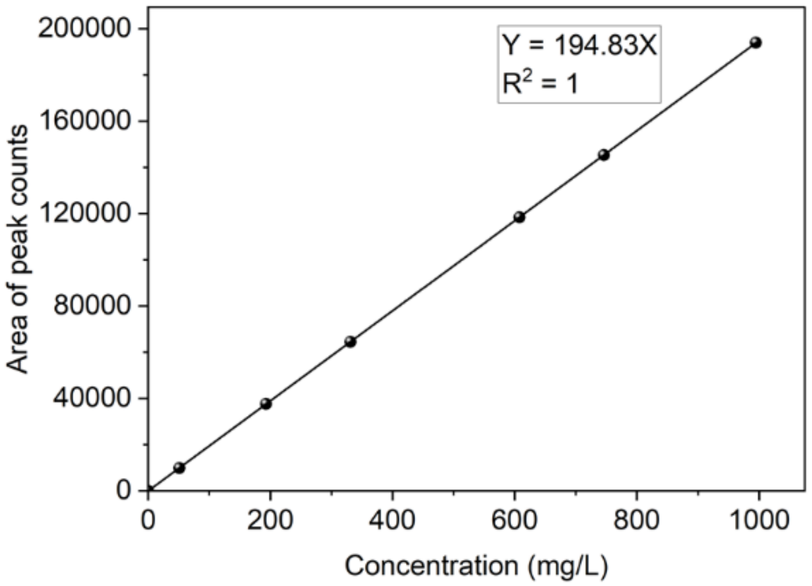

b)

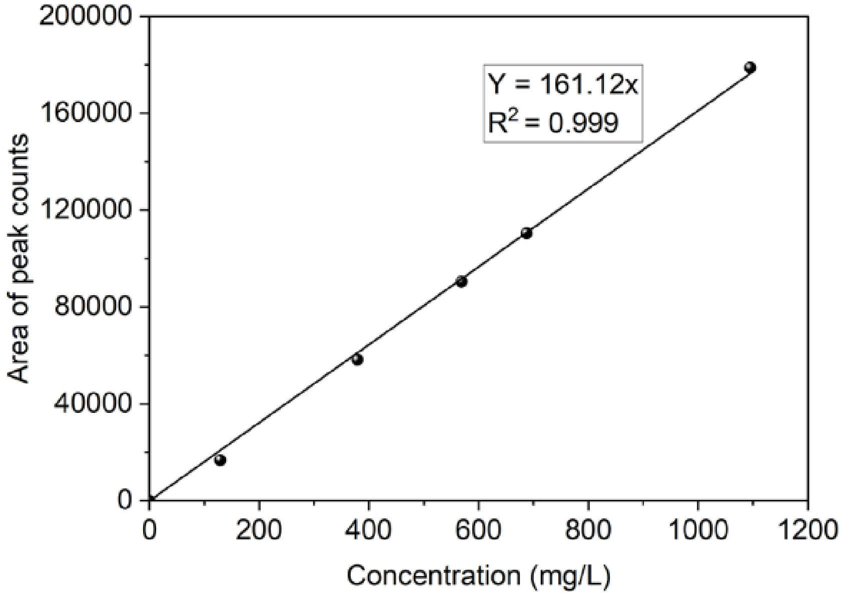

Figure S7: GC calibration curves of a) *N*-methyl-4-piperidone and b) *N*-methyl-2,3-dihydropyridin-4(1*H*)-one.

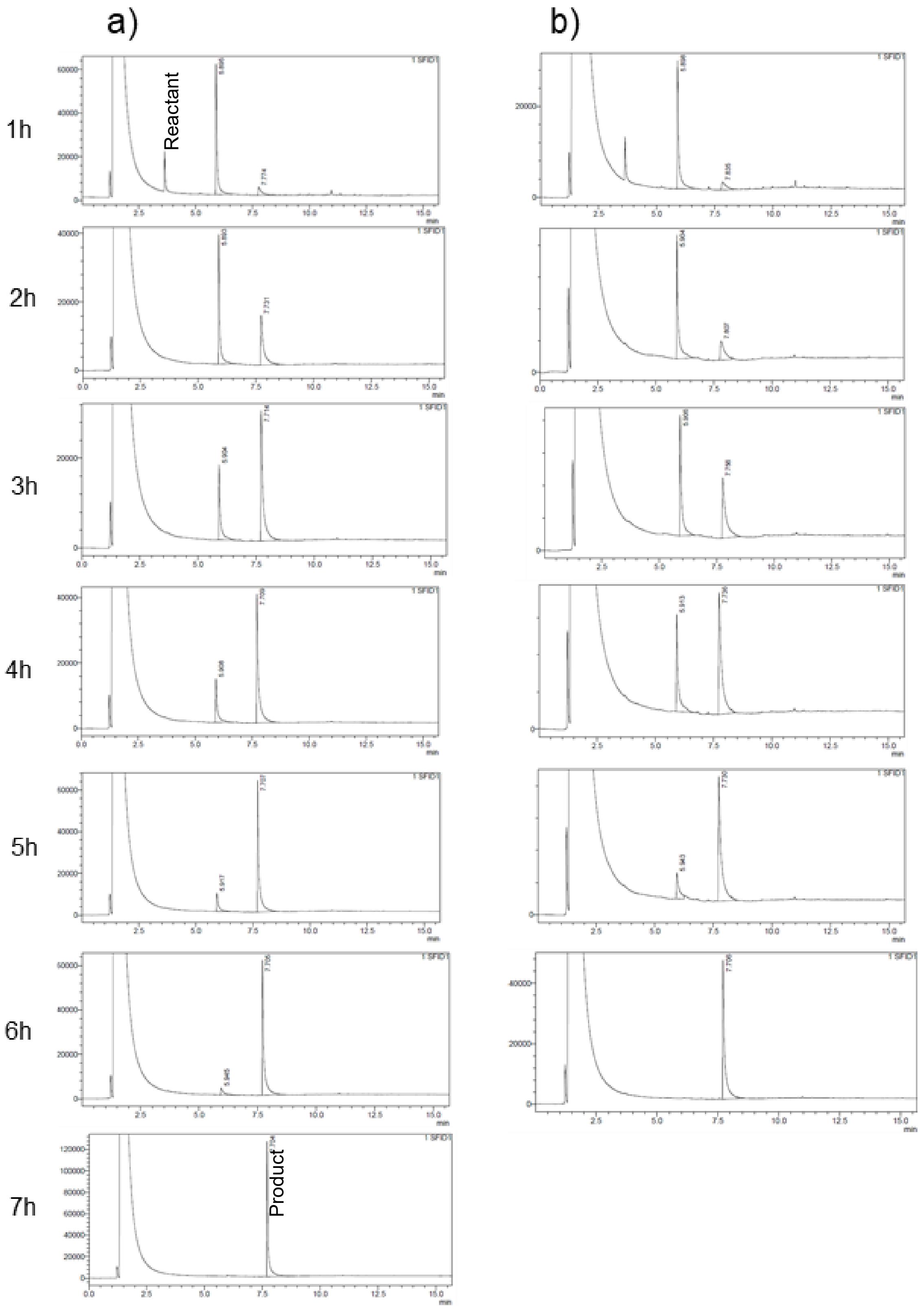

Figure S8: Monitoring the conversion of *N*-methyl-piperidone to *N*-methyl-2,3-dihydropyridin-4(1*H*)-one by GC over time with a) Au-Cit/rGO and b) Au-SiW9/rGO.

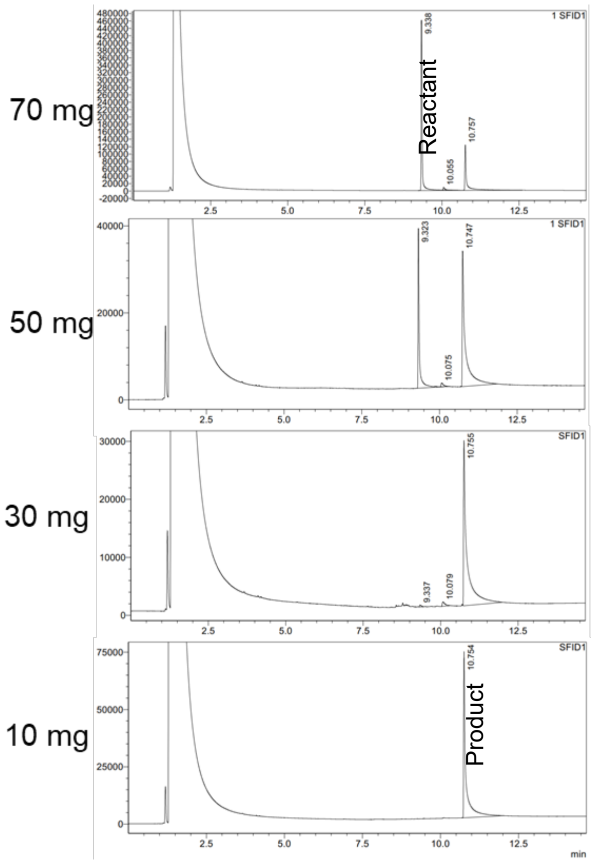

Figure S9: GC scan showing the influence of the amount of catalyst in α,β-ODH of *N*-benzyl-4-piperidone.

Gas Chromatography-Mass Spectrometry (GC-MS)

The sample was prepared by transferring 1 mL of the reaction mixture dissolved in acetone into a GC vial. The analysis was performed using the parameters given in the following Table S5.

Table S5: Conditions for GC-MS measurements.

Parameter	Value
Column	HP-5MS 30 m, 0.25 mm ID, 0.25 μm
Carrier gas	Helium (He), 2.0 mL/min
Injection	1 μL @ 100 μL/s, 250 °C
Split flow	20 mL/min
Split ratio	10
Detection	Agilent 5977E, Source : EI, FID
FID temperature	300 °C
Makeup gas	25 mL/min (Helium)
Gradient	0 min: 50 °C; 0.5 min: 50 °C; 30 °C/min to 300 °C; 2 min at 300 °C

The peak at 113.1 *m*/*z* in the mass spectrum in Figure S10 corresponds to the molecular ion of *N*-methyl-4-piperidone, consistent with its molecular formula $C_6H_{11}NO$ and a main isotope molecular weight of 113.16 g/mol. This peak is the most important indicator of the molecular identity.

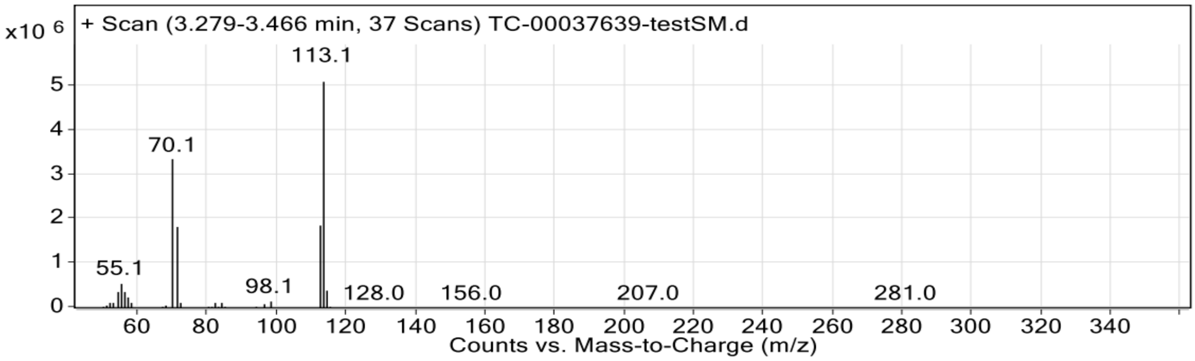

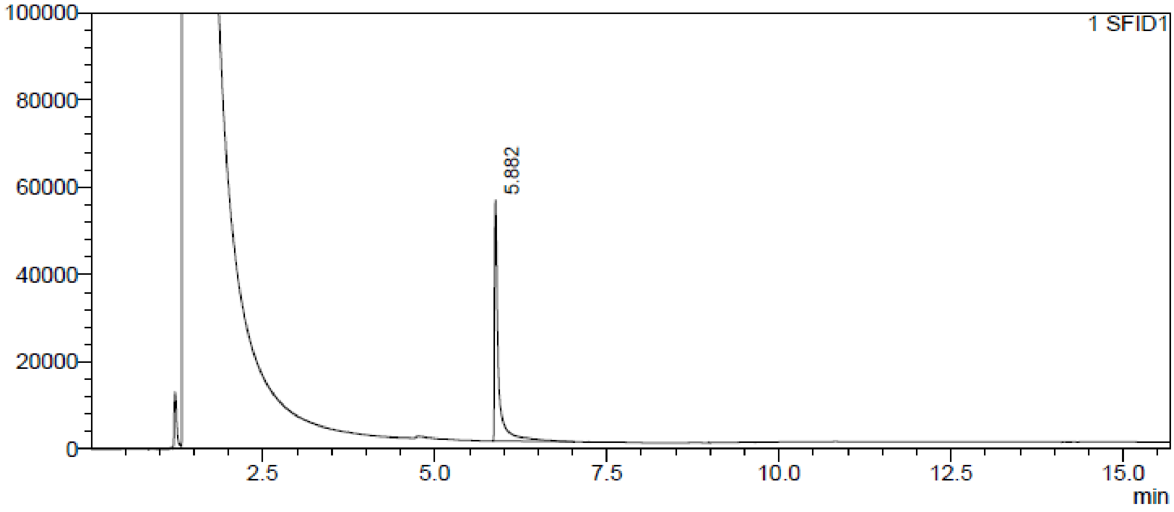

Figure S10: Mass spectrum and GC scan of *N*-methyl-4-piperidone.

The mass spectrum of the product in Figure S11 shows a prominent peak at 111.1 *m*/*z*, which confirms the removal of two hydrogen atoms from the starting material *N*-methyl-4-piperidone (molecular ion peak at 113.1 *m*/*z*). This decrease in molecular mass is consistent with the formation of a double bond during the ODH reaction, resulting in a molecular formula of C_6H_9NO. The presence of the 111.1 *m*/*z* peak provides supporting evidence of the successful dehydrogenation process and supports the structural changes observed in the ^{1}H NMR analysis.

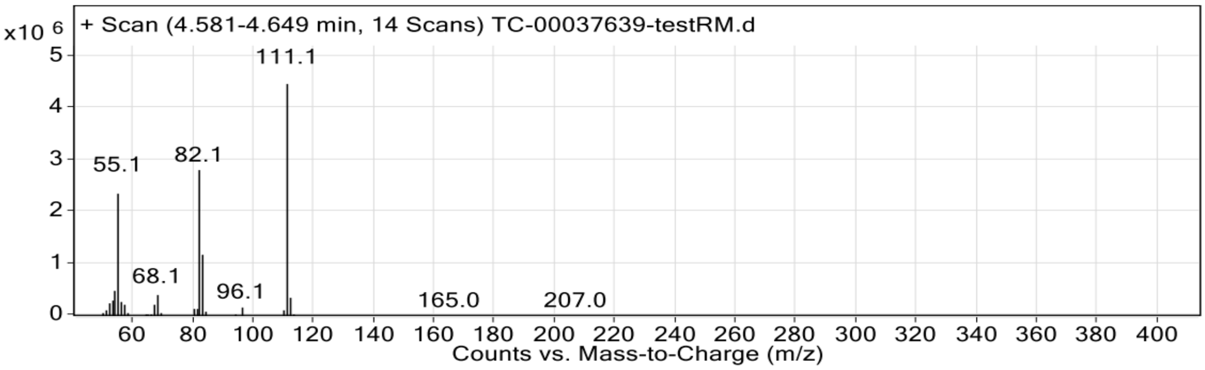

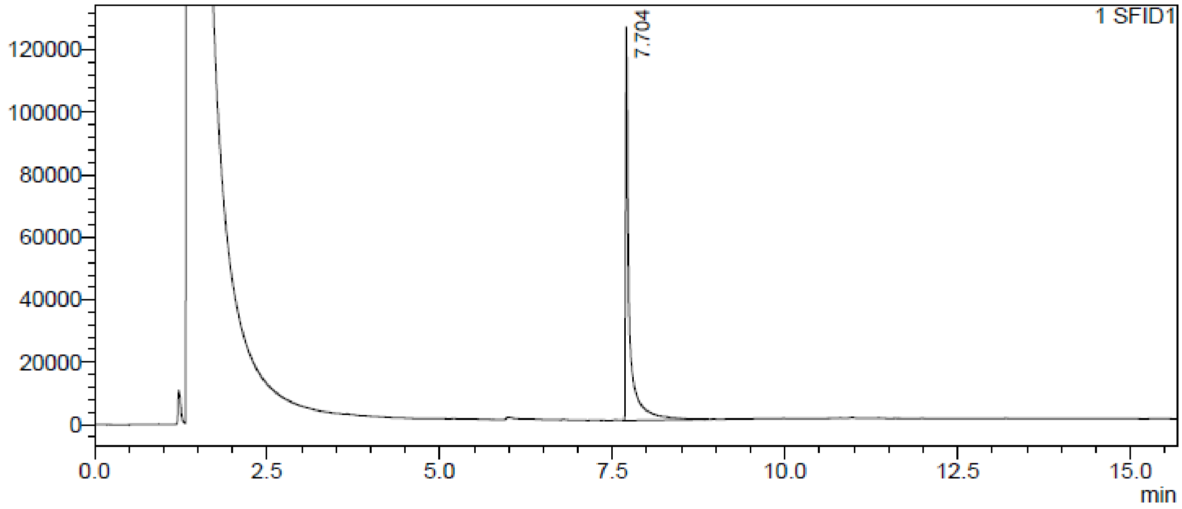

Figure S11: Mass spectrum of the reaction product *N*-methyl-2,3-dihydropyridin-4(1*H*)-one.

^{1}H NMR spectroscopy

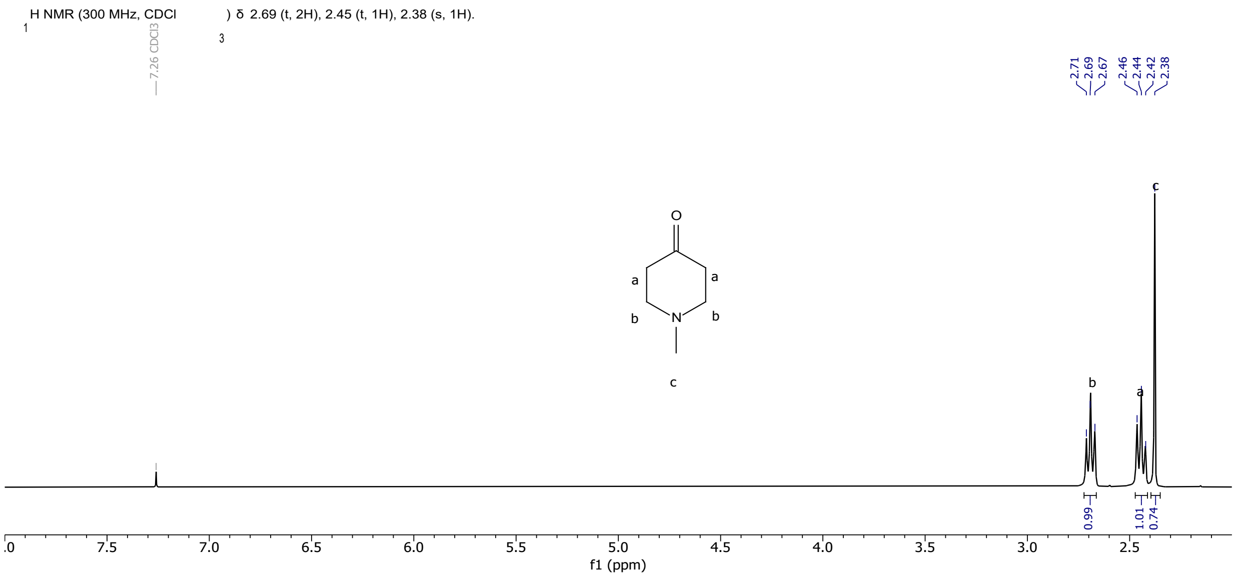

Figure S12: ^{1}H NMR spectrum (300 MHz) of *N*-methyl-4-piperidone in $CDCl_3$.

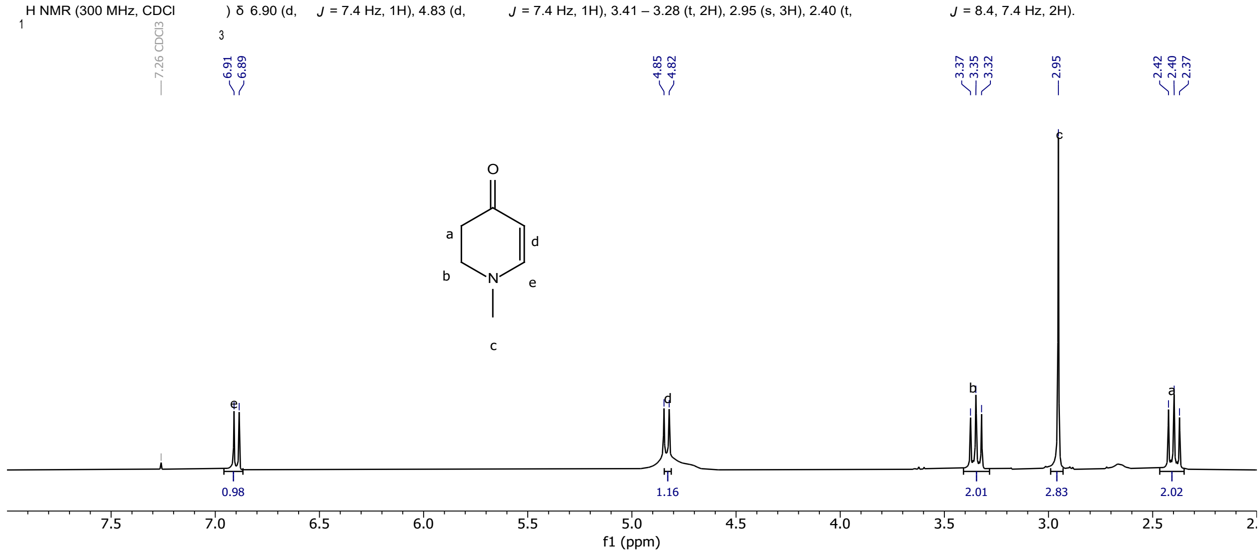

Figure S13: ^{1}H NMR spectrum (300 MHz) of the isolated product *N*-methyl-2,3-dihydropyridin-4(1*H*)-one in $CDCl_3$.

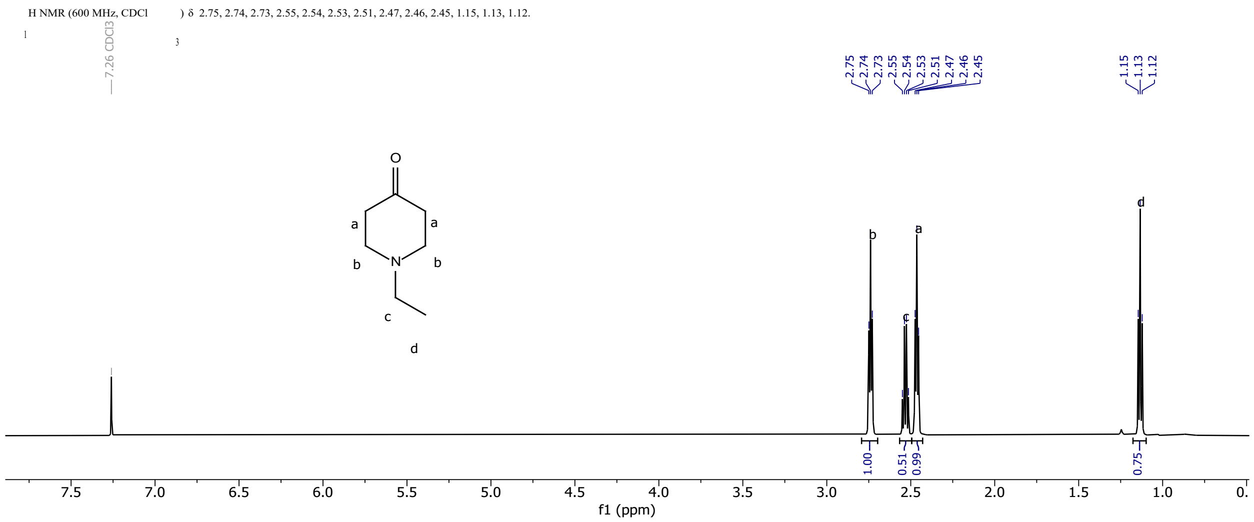

Figure S14: ^{1}H NMR spectrum (600 MHz) of *N*-ethyl-4-piperidone in $CDCl_3$.

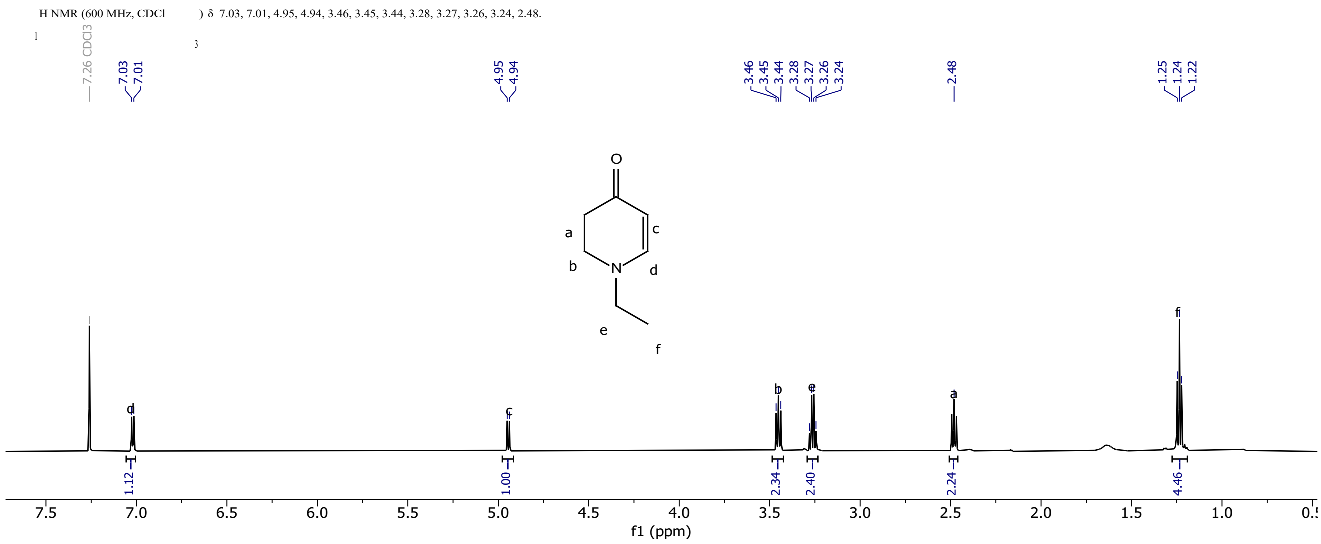

Figure S15: ^{1}H NMR spectrum (600 MHz) of the isolated product *N*-ethyl-2,3-dihydropyridin-4(1*H*)-one in $CDCl_3$.

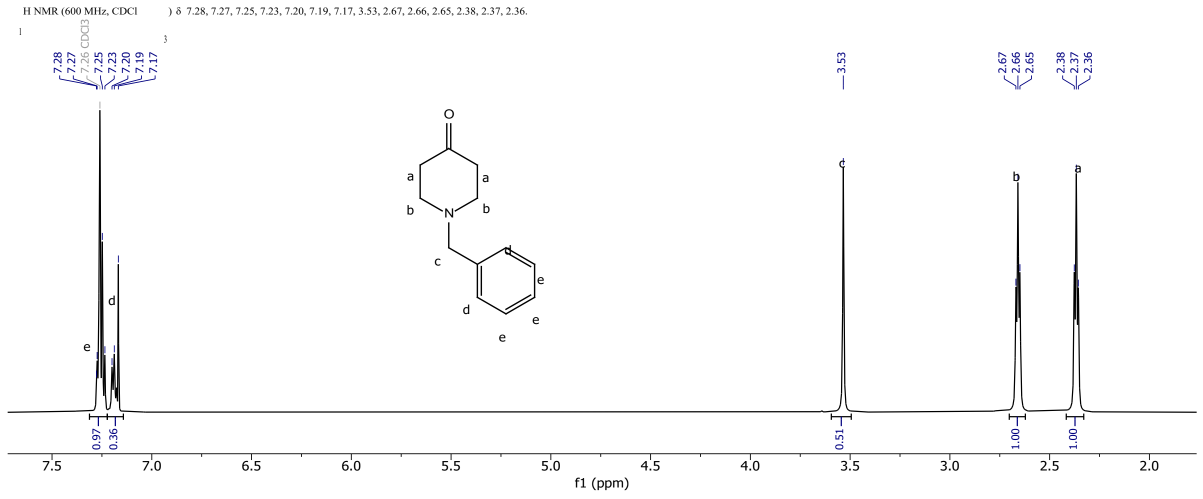

Figure S16: ^{1}H NMR spectrum (600 MHz) of *N*-benzyl-4-piperidone in $CDCl_3$.

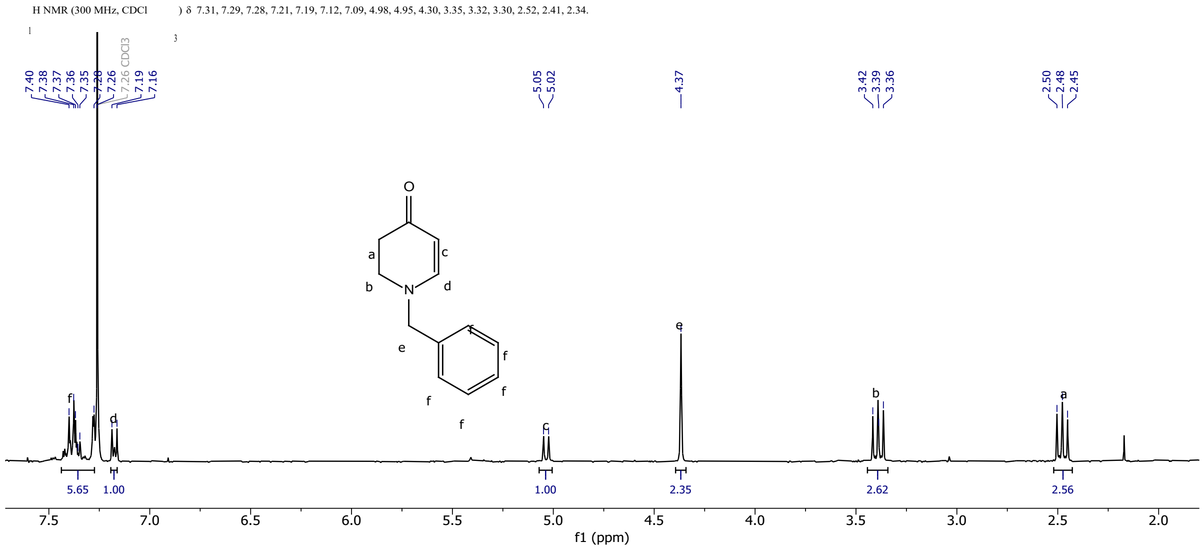

Figure S17: ^{1}H NMR spectrum (600 MHz) of the isolated product *N*-benzyl-2,3-dihydropyridin-4(1*H*)-one in $CDCl_3$.

Gold loading

To emphasize the effect of gold loading on the catalytic activity of the rGO and CB carbon-based catalyst, we compare the catalysts synthesized by reduction deposition (Table S6), where a lower amount of gold was deposited on the rGO and CB carbon support.

Preparation of Au-Cit/rGO, and Au-Cit/CB by RD method with lower gold loading

The synthesis procedures were the same as given in the main text, except that for rGO the amount of 500 mg (instead of 100 mg) and for CB the amount of 1000 mg (instead of 500 mg) was used. The amount of AC (1000 mg) remained the same.

Preparation of Au-SiW_9/rGO, and Au-SiW_9/CB by RD method with lower gold loading

The synthesis procedures were the same as given in the main text, except that for rGO the amount of 350 mg (instead of 50 mg) and for CB the amount of 700 mg (instead of 350 mg) was used. The amount of AC (700 mg) remained the same.

The results in Table S6 are to be compared with entries 2, 3, 5 and 6 in Table 1 in the main text.

Table S6: Catalytic results in the selective ODH of *N*-methyl-4-piperidone to *N*-methyl-2,3-dihydropyridin-4(1*H*)-one with lower gold loading for Au/rGO and Au/CB samples.[a]

Entry	Catalyst	Au (wt %)[b]	Catalyst (mg)[c]	Au/substrate[d]	Time (h)	Conversion (%)[e]	Yield (%)[f]
1	Au-Cit/rGO	2.08	10	0.0042	8	60	57
2	Au-Cit/CB	1.04	50	0.011	8	11	3
3	Au-SiW_9/rGO	0.85	10	0.0017	8	78	74
4	Au-SiW_9/CB	0.42	50	0.0043	8	25	12

[a] Reaction conditions: 30 mg, 0.25 mmol of *N*-methyl-4-piperidone, 2 mL of water, open air 1.013 bar, temperature 60 °C.

[b] Weight fraction of Au in the composite, calculated by assuming quantitative uptake of the AuNPs or the gold precursor onto the carbon material.

[c] Amount of applied composite in the catalysis. The amount was chosen so as to achieve the same molar fraction of Au in the reaction mixture of the three Au-X/carbon catalysts.

[d] mol Au / mol piperidone.

[e] Conversion was determined from the molar concentration of the starting material before the reaction minus the starting material after the reaction divided by the concentration of the starting material before (×100%).

[f] Yield was determined from the molar amount of the product divided by the used molar amount of the starting material (×100%).

Turnover number and turnover frequency

Table S7: TON (turnover number) and TOF (turnover frequency) of the rGO based catalyst determined at each hour of the ODH reaction of *N*-methyl-4-piperidone to *N*-methyl-2,3-dihydropyridin-4(1*H*)-one.

Time (h)	TON Au-Cit/rGO	TON Au-SiW_9/rGO	TON Au-SiW_9@rGO	TOF (h^{-1}) Au-Cit/rGO	TOF (h^{-1}) Au-SiW_9/rGO	TOF (h^{-1}) Au-SiW_9@rGO
1	29	25	22	290	25	22
2	60	41	35	300	21	18
3	86	65	60	29	22	20
4	92	80	75	23	20	19
5	99	102	93	20	20	19
6	108	118	106	18	20	18

TON = moles of product formed/moles of metal (of the catalyst), TOF = TON/time.
